# Insights into *Euphorbia* diversity: Probing the contrasts between *Euphorbia fischeriana* Steud and *Euphorbia ebracteolata* Hayata

**DOI:** 10.1016/j.jpha.2023.11.003

**Published:** 2023-11-28

**Authors:** Kaicheng Du, Yi Zhang, Lei Sun, Muke Tao, Tiantian Zuo, Yumeng Wang, Zhengfeng Zhang, Dali Meng

**Affiliations:** a School of Traditional Chinese Materia Medica, Shenyang Pharmaceutical University, Shenyang, 110016, China; b NMPA Key Laboratory for Quality Monitoring of Narcotic Drugs and Psychotropic Substances, Chongqing Institute for Food and Drug Control, Chongqing, 401121, China; c National Institutes for Food and Drug Control, Beijing, 100050, China

## Abstract

• First comparative study of *E. fischeriana* and *E. ebracteolate* by plant metabolomics was conducted.
• 29 characteristic metabolites were identified by UHPLC-QE-MS analysis.
• 8 constituents were identified as potential markers for discriminating two species.
• 12 representative compounds were isolated from two species.
• Two plants showed differences in antioxidant, antibacterial, and antitumor activities.

## Graphical abstract

**Figure:**
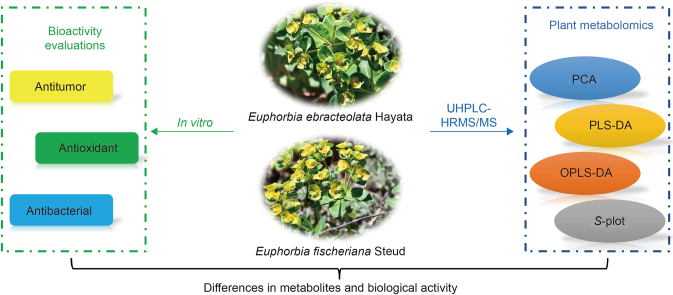

In traditional Chinese medicine (TCM)*, Euphorbia fischeriana* Steud (*E. fischeriana*) and *Euphorbia ebracteolata* Hayata (*E. ebracteolata*), commonly referred to as “Langdu”, are widely extensively utilized for treating lymphatic tuberculosis and ringworm [1]. Both plant species are perennial herbaceous plants mainly distributed in northeastern China, Mongolia, Russia (Siberia), and Republic of Korea [2]. There have been many reports on the chemical constituents and pharmacological effects of the two plant species, which has made more and more researchers realize that there may be differences between *E. fischeriana* and *E. ebracteolata*. In some cases, long-term improper use of herbal medicines can even lead to life-threatening conditions [3,4]. Therefore, it is essential to employ an effective technology to differentiate between these two plants based on their chemical constituents and biological activities, so as to reduce the harm caused by the mixing and misuse of medicinal materials. Therefore, the present paper describes a study of the differences between *E. ebracteolata* and *E. fischeriana*, using untargeted plant metabolomics and biological activity evaluations. This study aims to provide valuable insight into their equivalence and potential interchangeability in TCM and clinical medication.

To better understand the chemical constitutions of the *E. fischeriana* and *E. ebracteolata*, the primary and secondary metabolites were identified by the ultra-high-performance liquid chromatography-Q Exactive mass spectrometry (UHPLC-QE-MS) platform (Supplementary data). By comparing the MS/MS spectral data with the standard compounds, literature, and METLIN database, peaks 1–31 were identified and annotated, as presented in Table S1 [5]. Chemotaxonomically, the identified or annotated metabolites mainly belonged to terpenoids and acetophenone derivatives (Fig. 1A). Nearly half of the terpenoids are abietane diterpenoids, and the remaining consist of three atisane diterpenoids, one pimarane diterpenoid, three tigliane diterpenoids, one triterpenoid, and two unclassified diterpenes (Fig. 1B). Acetophenone derivatives include six acetophenone glycosides, two ordinary acetophenones, and one acetophenone dimer (Fig. 1C).

**Fig. 1:**
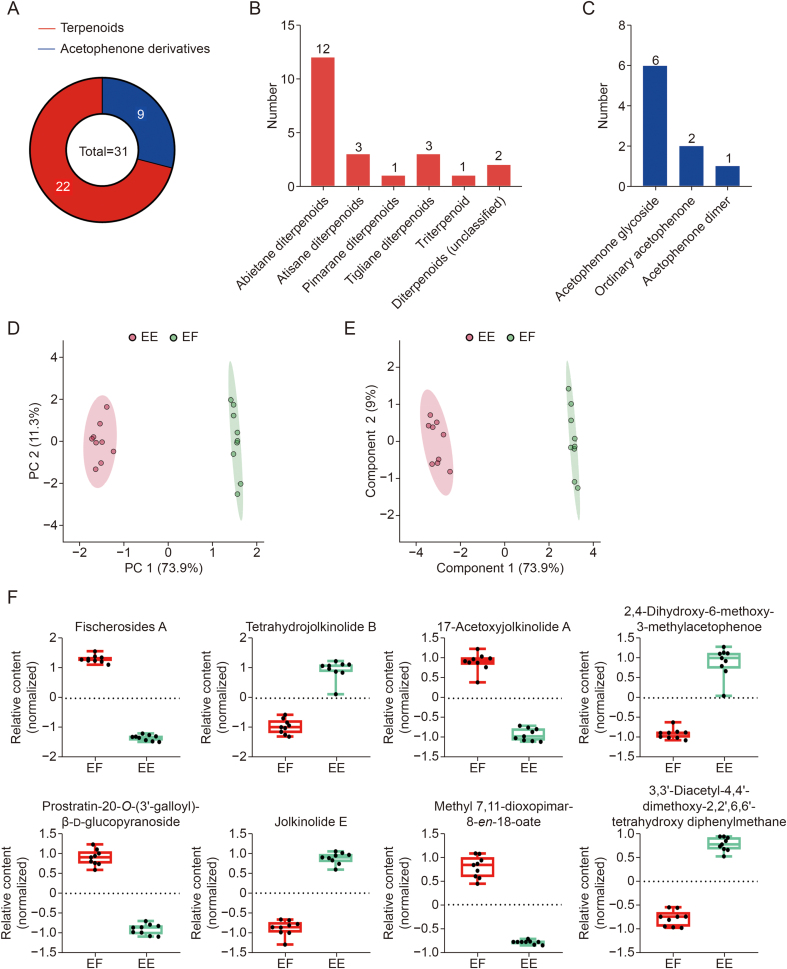
Results of plant metabolomics analysis. (A) Structural classification of the 31 metabolites identified. (B) Structural classification of terpenoids. (C) Structural classification of acetophenones. (D) Scores plot of principal component analysis (PCA) for liquid chromatography-mass spectrometry (LC-MS) metabolomics. (E) Scores plot of partial least-squares discriminant analysis (PLS-DA) for LC-MS metabolomics. (F) Relative content of eight metabolites that make the most significant contribution to distinguishing the two species. PC: principal component; EE: *Euphorbia ebracteolata* Hayata. EF: *Euphorbia fischeriana* Steud.

The identification results showed unique chemical components for each species. Therefore, we used untargeted plant metabolomics techniques to study the metabolite differences between them. As illustrated in Fig. 1D, samples were divided into two clusters on the PC1 (73.9 %) vs. PC2 (11.3 %) plane. In addition, to further obtain the metabolites that made important contributions to distinguish the two species, a partial least-squares discriminant analysis (PLS-DA) model was performed (Fig. 1E). When the top three components are generated, the *R*^2^ (cumulative) and *Q*^2^ (cumulative) are 0.985 and 0.972, respectively (Fig. S1), suggesting the applicability and predictability of the model. Similar results were also obtained from PLS-DA, two distinct clusters had variance values of 73.9% and 9% on component 1 and component 2, respectively. The difference in metabolites between the two species was also obvious by heatmap analysis (Fig. S2). Then, the variable importance value (VIP) and fold change (FC) value were calculated, and the key components were screened according to the condition of VIP > 1.2 and FC > 2.0 (Fig. S3 and Table S2). The relative contents of these markers are shown in Fig. 1F, among which peaks 12, 14, 13, and 28 are more abundant in *E. fischeriana*, and corresponding peaks 19, 15, 24, and 27 are more abundant in *E. ebracteolate*. According to the results of plant metabolomics, we found that the metabolic components of *E. fischeriana* and *E. ebracteolata* were significantly different, and selected eight key marker metabolites to distinguish the two species. In addition, the results of plant metabolomics on the same species in different regions indicated that different habitats have little effect on the composition of metabolites (Table S3 and Figs. S4 and S5).

A systematic phytochemical study was conducted to better understand the differences in chemical compositions and biological activities between the two plants. Twelve representative compounds (compounds **3**–**4** and **9**–**12** from *E. fischeriana*; compounds **1**–**2** and **5**–**8** from *E. ebracteolata*) were isolated from two plants using various chromatographic methods such as negative pressure silica gel, octadecylsilyl, Sephadex LH-20, and preparative and semi-preparative high performance liquid chromatography (HPLC) (Figs. S6–S31). Structurally, compounds **1**–**12** (Fig. 2A) could be mainly classified into three structural types, including acetophenone and acetophenone glycosides (compounds **1**–**5**), abietane diterpenoids (compounds **6**–**9**), and tigliane diterpenoids (compounds **10**–**12**). Compounds **5** and **9**–**11** were the iconic different metabolites of *E. fischeriana* and *E. ebracteolata*, selected by previous metabolomics research on plants. This work of extraction and isolation provided the material basis for further comparison of the biological activities of the two plants.

**Fig. 2:**
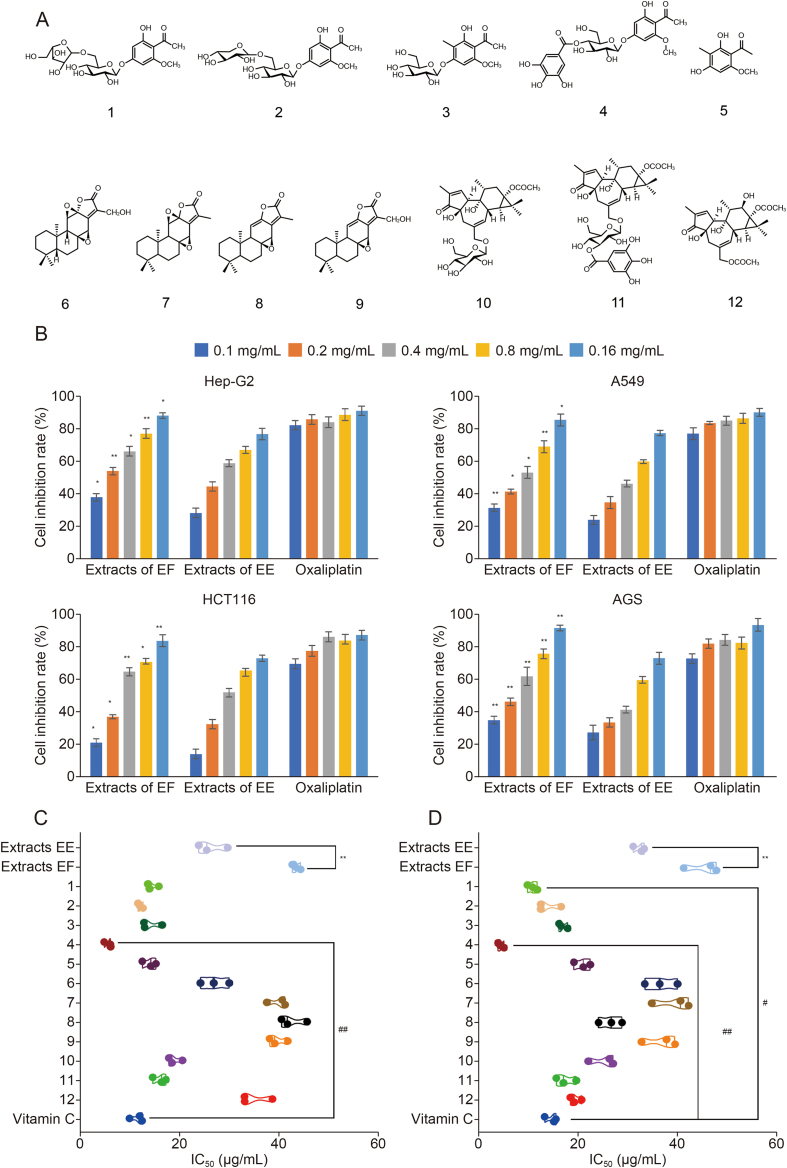
Results of phytochemical studies, antitumor, and antioxidant activities. (A) Chemical structures of compounds **1**–**12**. (B) Inhibitory effects of extracts on four human cancer cell lines: Hep-G2, A549, HCT116, and AGS). (C) The extracts and the compounds **1−12** in 2,2′-azino-*bis*(3-ethylbenzothiazoline-6-sulfonate) (ABTS) assays. (D) The extracts and the compounds **1−12** in 2,2-diphenyl-1-picrylhydrazyl (DPPH) assays. Data are presented as mean ± standard deviation (*n* = 3) and analyzed by analysis of variance. ^#^*P* < 0.05 and ^##^*P* < 0.01, vs. vitamin C; ^∗^*P* < 0.05 and ^∗∗^*P* < 0.01, extracts of *Euphorbia fischeriana* Steud (EF) vs. extracts of *Euphorbia ebracteolata* Hayata (EE) group; IC_50_: half maximal inhibitory concentration.

To clarify whether there is any difference in antitumor activity between *E. fischeriana* and *E. ebracteolata*, the inhibitory effects of compounds **1**–**12** and extracts against Hep-G2, A549, HCT116, and AGS were tested (Table 1 and Fig. 2B). The results of the antitumor activity test in this study showed that the extract of *E. fischeriana* exhibited stronger antitumor activity than those of *E. ebracteolate*. Combined with literature reports and results of compounds **1**–**12**, diterpenoids had the most significant antitumor activity. Metabolomics studies have shown that *E. fischeriana* contains more diterpenoids, especially tigliane diterpenoids, which may account for the difference in antitumor activity between the two species.

**Table 1:** Antitumor activity of compounds **1−12** against Hep-G2, A549, HCT116, and AGS cancer cells.

Compounds	IC_50_ values (μM)			
	Hep-G2	A549	HCT116	AGS
2-Hydroxy-6-methoxy-4-*O*-(6′-*O*-α-l-arabinofuranosyl-β-d-glucopyranosyl) acetophenone (compound **1**)	31.2 ± 4.2	50.9 ± 5.3	38.0 ± 3.1	42.1 ± 3.6
6-Hydroxyl-2-methoxyacetophenone-4-*O*-β-d-xylopyranosyl-(1→6)-β-d-glucopyranoside (compound **2**)	39.0 ± 4.0	39.1 ± 3.7	47.6 ± 5.5	31.4 ± 2.5
2,4-Dihydroxy-6-methoxy-3-methyl acetophenone-4-*O*-β-d-glucopyranoside (compound **3**)	32.1 ± 3.1	46.8 ± 4.9	50.2 ± 2.7	28.5 ± 1.4
2,4-Dihydroxy-6-methoxyacetophenone-4-*O*-(3′-galloyl)-β-d-glucopyranoside (compound **4**)	48.3 ± 1.6	34.4 ± 2.4	27.9 ± 2.8	27.6 ± 2.6
2,4-Dihydroxy-6-methoxy-3-methyl acetophenone (compound **5**)	31.9 ± 1.2	49.7 ± 2.7	41.4 ± 3.7	48.1 ± 5.6
17-Hydroxyjolkinolide B (compound **6**)	18.4 ± 2.7	14.3 ± 2.5	27.8 ± 1.9	13.9 ± 0.8
Jolkinolide B (compound **7**)	16.7 ± 3.4	20.2 ± 1.7	17.4 ± 1.1	18.7 ± 2.3
Jolkinolide A (compound **8**)	11.2 ± 1.6	12.0 ± 0.8	27.7 ± 2.3	17.7 ± 2.9
17-Hydroxyjolkinolide A (compound **9**)	9.3 ± 1.2	16.9 ± 1.3	19.1 ± 1.4	20.2 ± 2.6
Fischeroside A (compound **10**)	8.1 ± 0.2	9.7 ± 0.1	11.2 ± 1.5	4.5 ± 1.4
Prostratin 20-*O*-(3′-galloyl)-β-d-glucopyranoside (compound **11**)	6.7 ± 0.4	7.2 ± 0.8	9.2 ± 1.0	6.5 ± 1.1
Phorbol-13-acetate (compound **12**)	5.1 ± 0.7	10.2 ± 1.0	6.5 ± 1.3	2.7 ± 0.3
Oxaliplatin	6.9 ± 0.3	4.3 ± 0.7	5.7 ± 0.4	3.1 ± 0.4

IC_50_: half maximal inhibitory concentration (mean ± standard deviation).

Antioxidant activity plays a key role in studying the difference in pharmacological activity between *E. ebracteolate* and *E. fischeriana*. The free radical scavenging activities of compounds and extracts, along with vitamin C were evaluated by using 2,2′-azino-*bis*(3-ethylbenzothiazoline-6-sulfonate) (ABTS) and 2,2-diphenyl-1-picrylhydrazyl (DPPH) assay methods, respectively (Figs. 2C and D). Analyzing the results of the antioxidant activity of compounds **1**–**12**, it was found that acetophenone derivatives (compounds **1**–**5**) have the strongest antioxidant capacity, which also suggested that they were the key secondary metabolites with significant antioxidant activity of the two species. In addition, the experimental results showed that extracts of *E. ebracteolata* had stronger antioxidant activity than extracts of *E. fischeriana*.

The antibacterial activity of compounds **1**–**12** and extracts were tested employing Gram-positive bacteria *Staphylococcus aureus* (*S. aureus*) and *Bacillus subtilis* (*B. subtilis*) and Gram-negative bacteria *Escherichia coli* (*E. coli*) and *Pseudomonas aeruginosa* (*P.* *aeruginosa*), respectively (Table 2 and Fig. S32). Berberine and penicillin were selected as positive drugs. From the minimum inhibitory concentration (MIC) results of compounds **1**–**12**, it could be seen that the abietane diterpenoids, the common component of the two plants, have the most significant antibacterial activity, which indicated that abietane diterpenes may be the material basis for their antibacterial activity. Further experimental results showed the extracts of *E. fischeriana* and *E. ebracteolata* showed strong antibacterial activity, which also verified the above conclusions. More detailed analysis and discussion of the activity results are shown in the Supplementary data.

**Table 2:** Minimum inhibitory concentration (MIC) of compounds **1**−**12** and berberine, and penicillin on *Staphylococcus aureus* (*S. aureus*)*, Bacillus subtilis* (*B. subtilis*), *Escherichia coli* (*E. coli*), and *Pseudomonasaeruginosa* (*P.aeruginosa*).

Compounds	MIC (μg/mL)			
	*S. aureus*	*B. subtilis*	*E. coli*	*P.aeruginosa*
2-Hydroxy-6-methoxy-4-*O*-(6′-*O*-α-l-arabinofuranosyl-β-d-glucopyranosyl) acetophenone (compound **1**)	12.5	25.00	12.50	12.50
6-Hydroxyl-2-methoxyacetophenone-4-*O*-β-d-xylopyranosyl-(1→6)-β-d-glucopyranoside (compound **2**)	12.50	6.25	6.25	12.50
2,4-Dihydroxy-6-methoxy-3-methyl acetophenone-4-*O*-β-d-glucopyranoside (compound **3**)	25.00	12.50	12.50	6.25
2,4-Dihydroxy-6-methoxyacetophenone-4-*O*-(3′-galloyl)-β-d-glucopyranoside (compound **4**)	12.50	12.50	3.12	6.25
2,4-Dihydroxy-6-methoxy-3-methyl acetophenone (compound **5**)	6.25	12.50	6.25	3.12
17-Hydroxyjolkinolide B (compound **6**)	12.50	6.50	3.12	3.12
Jolkinolide B (compound **7**)	6.50	12.50	0.78	1.56
Jolkinolide A (compound **8**)	6.50	6.50	1.56	1.56
17-Hydroxyjolkinolide A (compound **9**)	6.50	3.12	1.56	0.78
Fischeroside A (compound **10**)	>50.00	>50.00	50.00	>50.00
Prostratin 20-*O*-(3′-galloyl)-β-d-glucopyranoside (compound **11**)	>50.00	>50.00	>50	25.00
Phorbol-13-acetate (compound **12**)	>50.00	25.00	25.00	25.00
Berberine	1.56	6.50	3.12	6.50
Penicillin	0.78	1.56	1.56	3.12

In this study, non-targeted metabolomics techniques were used to analyze the metabolite differences between *E. ebracteolata* and *E. fischeriana*, which were not obvious by the simple analysis of phytochemical research and chromatograms. In addition, the difference in antibacterial, antitumor, and antioxidant activities of the two species was evaluated. These results showed that *E. ebracteolata* and *E. fischeriana* not only have significant differences in chemical composition but also show different effects in antibacterial, antitumor, and antioxidant. In summary, this study demonstrated that the mixture and misuse of *E. ebracteolata* and *E. fischeriana* in industrial production and clinical use should be avoided. Moreover, this study demonstrated the efficacy of integrating metabolomic and bioactive strategies for distinguishing medicinal materials with similar appearances and morphologies, ultimately providing promising prospects in the identification and utilization of medicinal plants.

## CRediT author statement

**Kaicheng Du:** Methodology, Validation, Formal analysis, Investigation, Data curation, Writing - Reviewing and Editing, Visualization; **Yi Zhang:** Writing - Reviewing and Editing, Formal analysis, Visualization; **Lei Sun:** Conceptualization, Methodology; **Muke Tao:** Formal analysis, Investigation, Data curation; **Tiantian Zuo:** Validation, Data curation; **Yumeng Wang:** Writing - Reviewing and Editing; **Zhengfeng Zhang:** Formal analysis, Investigation; **Dali Meng:** Resources, Writing - Reviewing and Editing, Supervision, Funding acquisition.

## Declaration of competing interest

The authors declare that there are no conflicts of interest.
