# Supplementary material for: Insights into Euphorbia diversity: Probing the contrasts between Euphorbia fischeriana Steud and Euphorbia ebracteolata Hayata
Source: J Pharm Anal. 2023 Nov 28;14(5):100896. doi: 10.1016/j.jpha.2023.11.003 (PMC11106527; doi:10.1016/j.jpha.2023.11.003)
Supplement: Multimedia component 1 [file mmc1.docx]

**Supplementary Material**

**Insights into *euphorbia* diversity: probing the contrasts between *Euphorbia fischeriana* Steud and *Euphorbia ebracteolata* Hayata**

**Table of Contents**

[**1. Material and methods** 4](#_Toc146310832)

[1.1. Plant material 4](#_Toc146310833)

[1.2. Sample preparation for UHPLC-QE-MS metabolomics 4](#_Toc146310834)

[1.3. UHPLC-QE-MS analysis 4](#_Toc146310835)

[1.4. Data Processing 5](#_Toc146310836)

[1.5. Phytochemical research 5](#_Toc146310837)

[1.6. Antitumor activities 6](#_Toc146310838)

[1.7. Antioxidant activities 7](#_Toc146310839)

[1.8. Antibacterial assays 7](#_Toc146310840)

[1.9. statistical analysis 8](#_Toc146310841)

[**2. Results and discussion** 8](#_Toc146310842)

[**3. Tables** 11](#_Toc146310843)

[Table. S1 Identification of mass features detected in the extracts of *E. fischeriana* and *E. ebracteolata*. 11](#_Toc146310844)

[Table. S2 The key components were screened according to the condition of variable importance value (VIP) > 1.2 and fold change (FC) > 2.0. 14](#_Toc146310845)

[Table. S3 Collecting information of 18 batches of different varieties of *E. fischeriana* and *E. ebracteolata*. 14](#_Toc146310846)

[**4. Figures** 16](#_Toc146310847)

[Fig. S1 The partial least-squares discriminant analysis (PLS-DA) model overview of different numbers of principal components 16](#_Toc146310848)

[Fig. S2 The heat map of metabolites **1−31** from both the species. EE: *Euphorbia ebracteolata* Hayata*.* EF: *Euphorbia fischeriana* Steud. 16](#_Toc146310849)

[Fig. S3 Chemical markers with variable importance value (VIP)values larger than 1.2. EE: *Euphorbia ebracteolata* Hayata*.* EF: *Euphorbia fischeriana* Steud. 17](#_Toc146310850)

[Fig. S4 The principal component analysis (PCA) Scores plot of *Euphorbia fischeriana* Steud 17](#_Toc146310851)

[Fig. S5 The principal component analysis (PCA) Scores plot of *Euphorbia ebracteolata Hayata* 18](#_Toc146310852)

[Fig. S6 The isolation procedures of *Euphorbia fischeriana* Steud 18](#_Toc146310853)

[Fig. S7 The isolation procedures of *Euphorbia ebracteolata Hayata* 19](#_Toc146310854)

[Fig. S8 The ^1^H-NMR spectrum of Comp. **1** 19](#_Toc146310855)

[Fig. S9 The ^13^C-NMR spectrum of Comp. **1** 20](#_Toc146310856)

[Fig. S10 The ^1^H-NMR spectrum of Comp. **2** 20](#_Toc146310857)

[Fig. S11 The ^13^C-NMR spectrum of Comp. **2** 21](#_Toc146310858)

[Fig. S12 The ^1^H-NMR spectrum of Comp. **3** 21](#_Toc146310859)

[Fig. S13 The ^13^C-NMR spectrum of Comp. **3** 22](#_Toc146310860)

[Fig. S14 The ^1^H-NMR spectrum of Comp. **4** 22](#_Toc146310861)

[Fig. S15 The ^13^C-NMR spectrum of Comp. **4** 23](#_Toc146310862)

[Fig. S16 The ^1^H NMR spectrum of Comp. **5** 23](#_Toc146310863)

[Fig. S17 The ^13^C NMR spectrum of Comp. **5** 24](#_Toc146310864)

[Fig. S18 The ^1^H-NMR spectrum of Comp. **6** 24](#_Toc146310865)

[Fig. S19 The ^13^C-NMR spectrum of Comp. **6** 25](#_Toc146310866)

[Fig. S20 The ^1^H NMR spectrum of Comp. **7** 25](#_Toc146310867)

[Fig. S21 The ^13^C NMR spectrum of Comp. **7** 26](#_Toc146310868)

[Fig. S22 The ^1^H NMR spectrum of Comp. **8** 26](#_Toc146310869)

[Fig. S23 The ^13^C NMR spectrum of Comp. **8** 27](#_Toc146310870)

[Fig. S24 The ^1^H NMR spectrum of Comp. **9** 27](#_Toc146310871)

[Fig. S25 The ^13^C NMR spectrum of Comp. **9** 28](#_Toc146310872)

[Fig. S26 The ^1^H-NMR spectrum of Comp. **10** 28](#_Toc146310873)

[Fig. S27 The ^13^C-NMR spectrum of Comp. **10** 29](#_Toc146310874)

[Fig. S28 The ^1^H-NMR spectrum of Comp. **11** 29](#_Toc146310875)

[Fig. S29 The ^13^C-NMR spectrum of Comp. **11** 30](#_Toc146310876)

[Fig. S30 The ^1^H-NMR spectrum of Comp. **12** 30](#_Toc146310877)

[Fig. S31 The ^13^C-NMR spectrum of Comp. **12** 31](#_Toc146310878)

[Fig. S32 Inhibitory rate of extracts from *Euphorbia fischeriana* Steud (EF) and *Euphorbia ebracteolata* Hayata (EE), berberine, and penicillin on *S. aureus, B. subtilis*, *E. coli* and *P. Aeruginosa* at 500 μg/mL. Data are presented as mean ± SD (n = 3) and analyzed by ANOVA. *P < 0.05 and **P < 0.01 compared with berberine group, ^#^P< 0.05 compared with penicillin group. 31](#_Toc146310879)

[**5. References** 32](#_Toc146310880)

**1. Material and methods**

1.1. Plant material

Plant material used for plant metabolic analysis of *Euphorbia fischeriana* Steud and *Euphorbia ebracteolata* Hayata were collected from different habitats in China (**Table. S1**). In addition, the plant material used for separation and pharmacological test were again collected in large quantities from Anhui Province, China. All of them were authenticated by Prof. Jincai Lu (Shenyang Pharmaceutical University) based on literature and morphology. Specimens of representative vouchers (DJ-2021-1-20) were placed in the School of Traditional Chinese Material Medica at Shenyang Pharmaceutical University.

1.2. Sample preparation for UHPLC-QE-MS metabolomics

First, the air-dried roots of two plants, collected from different habitats, were pulverized into fine powder using a mill. Each sample (1.0 g, fine powder) was accurately weighed and extracted with 25 mL of methanol in an ultrasonic bath at 100 Hz for 30 min. After centrifuged, the supernatants were filtered through a 0.22 *μ*m membrane filter and the filtrate was stored at 4°C as a sample solution until UHPLC-MS analysis. The quality control sample, a mixture of aliquots from all samples, was prepared to assess instrument stability. The samples of quality control were examined three times initially and injected every 6 times during metabolite analysis.

1.3. UHPLC-QE-MS analysis

The analyses of untargeted metabolite profiling were performed on an Ultimate 3000 UHPLC system coupled to Q Exactive MS (Thermo Fisher Scientific, CA, USA). Chromatographic column: Waters Acquity UHPLC BEH C_18_ column (2.1 × 100 mm, 1.7 *μ*m). The mobile phase was composed of (A) water with 0.1% formic acid and (B) acetonitrile. The gradient began with 90% A and 10% B, 1-8 min, ramping to 75% A and 25% B, 8-15 min, 65% A and 35% B, 15-20 min, 50% A and 50% B, 20-25 min, 25% A and 75% B, 25-30 min, 10% A and 90% B, 30-39 min. The column temperature was adjusted to 30°C, and the flow rate was kept at 0.3 mL/min. The MS analysis of lipids was carried out on Q Exactive under the conditions: Sheath gas flow rate:45, Aux gas flow rate: 10, Spray voltage (kv): 3.00 (negative), 3.80 (positive), Capillary temp: 320°C, S-lens RF level: 50.0, Source temp: 300°C, runtime: 1.5-39 min; Full MS: Resolution: 70000, AGC target: 1e6, Maximum IT: 100 ms, scan range: 100-1200 *m/z*; dd-MS2: Resolution: 17500, AGC target: 1e5, Maximum IT: 50 ms, loop count: 5, isolation window: 4.0 *m/z*, NCE: 15, 35, 45.

1.4. Data Processing

The LC-HRMS data files (.raw) were uploaded to the XCMS Online software platform (https://xcmsonline.scripps.edu/). The XCMS preprocessing transformed the raw data into a table of retention time, peak picking, mass feature intensity, *m/z* values and annotation. Import the data from XCMS Online into MetaboAnalyst 5.0 Online (https://www.metaboanalyst.ca/) and normalize the data, including logarithmic transformation and Pareto scaling. Then the normalized data is further analyzed to perform the multivariate data analysis including principal components analysis (PCA), partial least squares-discriminant analysis (PLS-DA) and heatmap. The statistical properties of the models were evaluated by R^2^ and Q^2^.

1.5. Phytochemical research

The instruments used in the study of phytochemistry included Bruker AVANCE III HD (Bruker, Fällanden, Switzerland), LC-20AD pump and SPD-20A ultraviolet detector (SHIMADZU, Kyoto, Japan). Materials used for separation included HPLC C18 silica gel, HPLC Pbr silica gel (Nacalai tesque, Japan), open column silica gel (Qingdao Haiyang Chemical Group Corporation, Qingdao, China) and Sephadex LH-20 (Green Herbs Science and Technology Development Co., Ltd. China). All solvents were HPLC grade (Concord Technologies Company, Ltd., Tianjin, China).

The air-dried root of *E. fischeriana* (5 kg) and *E. ebracteolata* (5 kg) were extracted with methanol (20 L) under reflux for 2 times (60 ℃, 3 h, 2 h). The solvent was refluxed under reduced pressure to obtain 209 g (*E. fischeriana*) and 147 g (*E. ebracteolata*) extracts, respectively. The extract of *E. fischeriana* (209 g) was subjected to separation over a silica gel, eluting with a gradient system of CH_2_Cl_2_/MeOH (100:2 to 0:100) to obtain seven fractions (Fr. 1-7). Fr. 3 (9.5 g) was further fractionated over open column ODS, using MeOH/H_2_O (0:100-100:0) as the elution system to give five fractions (Fr. 3.1-3.5). Fr 3.3 (1.7 g) was separated *via* HPLC (CH_3_OH/H_2_O 67:33), resulting in **11** (45.7 mg) and **10** (21.1 mg). Fr 3.4 (0.7 g) was separated *via* HPLC (CH_3_OH/H_2_O 80:20), resulting in **12** (10.4 mg). Fr. 2 (18.4 g) was further fractionated over ODS, using MeOH/H_2_O (25:75, 50:50 75:25, 100:0) as the elution system to give five fractions (Fr. 2.1-2.4). Compound **9** (151.7 mg) was isolated from Fr. 2.2 (1.4) by Sephadex LH-20. Fr. 5 (6.8 g) was further fractionated over ODS with MeOH/H_2_O (0:100-100:0) as the elution system to give five fractions (Fr. 5.1-5.5). Fr 5.2 (0.9 g) was separated *via* HPLC (CH_3_OH/H_2_O 40:60), resulting in **3** (31.1 mg) and **4** (55.3 mg). Similarly, the extract of *E. ebracteolata* (147 g) was subjected to separation over a silica gel, eluting with a gradient system of CH_2_Cl_2_/MeOH (100:0 to 100:100) to obtain five fractions (Fr. 1-5). Fr. 2 (11.5 g) and 3 (9.1 g) were combined and further fractionated over ODS, using MeOH/H_2_O (50:50-100:0) as the elution system to give three fractions (Fr. 2.1-2.3). Fr. 2.1 (2.7 g) was further separated via HPLC (CH_3_OH/H_2_O, 50:50, *v/v*) to obtain **6** (17.4 mg). Fr. 2.2 (2.7 g) was further separated via HPLC (CH_3_OH/H_2_O, 60:40, *v/v*) to obtain **8** (29.4 mg) and **7** (36.6 mg). Fr. 4 (21.4 g) was further fractionated over ODS with MeOH/H_2_O (20:80-80:20) as the elution system to give three fractions (Fr. 4.1-4.4). Fr. 4.2 (3.1 g) was further separated *via* HPLC (CH_3_OH/H_2_O, 31:39, *v/v*) to obtain **2** (61.4 mg), **1** (24.4 mg) and **5** (20.2 mg). The flow charts of the separation of the two plants are in the supplementary materials.

1.6. Antitumor activities

Antitumor activities of the extracts and compounds were evaluated against several human cancer cell lines, including Hep-G2, A549, HCT116, and AGS (obtained from ATCC, Rockville, MD, USA) by using CCK-8 assay. Oxaliplatin was used as positive controls. Samples (extracts, compounds, and positive controls) were dissolved in medical DMSO and diluted with complete medium to various concentrations. The tumor cell lines were maintained in 5% CO_2_ at 37°C. When the cells entered the logarithmic phase, cells were prepared into cell suspension and inoculated into 96-well plates with 1×10^4^ cells per well and 3 parallel wells. After cells attachment, the cells were pretreated with the samples in different concentrations for 24 h. Then, 10 *μ*L CCK-8 was added to the cells and incubated for 2-4 h. Absorbance was then measured by a microplate reader at 450 nm.

1.7. Antioxidant activities

The scavenging capacities of tested samples on free radicals including ABTS, DPPH were detected according to the corresponding instruction manual and with slight modifications [1]. Vitamin C were employed as positive control. Samples and Vitamin C were dissolved in absolute ethanol and prepared to get six concentrations (3.125, 6.25, 12.5, 25, 50 and 100 μg/mL).

100 μL of ethanol solution containing 2×10^−4^ mol/L DPPH was added to 100 μL of sample solution with a certain concentration. The mixed solution was shaken well before reacting for 30 min at room temperature in darkness. Optical density (OD) value was measured with a microplate reader at 517 nm. The DPPH solution was replaced by an equal volume of absolute ethanol as the blank group. Every group was parallelly designed 3-well repeat.

ABTS radical cation was produced by reacting 14 mM stock solution of ABTS with 4.9 mM potassium persulphate in a volume ratio of 1:1. The reaction mixture was used after standing at room temperature in the dark for 12 hours. Before the detection, the ABTS+ solution was diluted with ethanol to an absorbance of 0.7 at 734 nm. The 20 μL samples at various concentrations were mixed with 180 μL ABTS +·working solution and the reaction mixture was left to incubate for 15 min at room temperature, and then was measured with a microplate reader at 734 nm.

Then, all the results were expressed in terms of the IC_50_ values (*μ*g/mL), i.e., the 50% scavenging effect concentration.

1.8. Antibacterial assays

Briefly, antibacterial activities of the extracts and compounds were assessed against both Gram-positive/negative bacteria including *Staphylococcus aureus* (CMCC B26003), *Bacillus subtilis* (CMCC B63501), *Escherichia coli* (CMCC B44102) and *Pseudomonas aeruginosa* (CMCC B10104). The bacterial were incubated in Luria-Bertani (LB) medium at in 37°C for 24 h. Then the bacterial suspensions were adjusted with medium to 1 ×10^5^ CFU/mL for the next tests. Stock solutions of the tested extracts and positive drug were prepared in DMSO and were diluted to 500 μg/mL with LB medium. Bacterial suspension (100 *μ*L) and the samples solution (100 *μ*L) were added to each well of a 96-well microtiter plate in triplicate to be incubated for 24 h at 37°C. Absorbance was then measured by a microplate reader at 600 nm. Where OD_1_, OD_2_ and OD_3_ were the absorbencies of the control, sample and blank, respectively. Similarly, stock solutions of the tested compounds and positive drug were prepared in DMSO. Each stock solution was 2-fold diluted with LB medium to give serial concentrations from 200 to 0.38 μg/mL. Bacterial suspension (100 *μ*L) and the samples solution (100 *μ*L) were added to each well of a 96-well microtiter plate in triplicate to be incubated for 24 h at 37°C. By visual observation, the MIC of the tested bacteria was those with no bacterial growth in the tube of the lowest concentration of the sample. Inhibition (%) = $\frac{\text{OD}_{\text{1}}\text{-}\text{OD}_{\text{2}}}{\text{OD}_{\text{1}}\text{-}\text{OD}_{\text{3}}}\text{×100\%}$.

1.9. statistical analysis

The IC_50_ values for the extracts and compounds **1-12** were calculated with SPSS software package. The significant differences of data were analyzed by one-way analysis of variance (ANOVA) followed by the Dunnett’s multiple comparison tests using Graphpad Prism version 8.01.

**2. Results and discussion**

For the scientific development of *E. fischeriana* and *E. ebracteolate*, extensive phytochemical studies have been carried out on these two medicinal plants, and a large number of small molecular natural products have been found, including triterpenoids, diterpenoids, flavonoids, acetophenones, tannins, etc [2-4]. Under the treatment of XCMS Online, the valid mass features of Mass spectrometry data were selected for the identification of the compositions. Peaks 1-31 were identified and annotated, as presented in **Table S1** [5-26], including retention time, experimental *m/z*, origin, formula, main MS/MS fragments, and so on. These levels of metabolite identification, as were recommended by [27] and [28] were categorized into three levels. Level 1: identified compounds (comparison with standards); Level 2: putatively annotated compounds (compared with literature and Metlin (http://metlin.scripps.edu) database); Level 3: putatively characterized compound classes. This included retention time, experimental *m/z*, origin, formula, main MS/MS fragments, and so on.

As a development of traditional applications, the application of *E. fischeriana* and *E. ebracteolate* in antitumor has been greatly developed [29, 30]. Whether there are different in antitumor activities between the them is one of the key factors affecting their clinical application. Firstly, the antitumor activity of the compounds **1−12** was also tested against four cancer cell lines. The results are shown in Table 2. compounds **10−12** showed strongest antitumor activities with IC_50_ values ranging from 2.7 to 11.2 μM, compounds **6−9** showed medium antitumor activities with IC_50_ values ranging from 9.3 to 27.8 μM, compounds **1−5** showed weakest antitumor activities (IC_50_, 27.6-50.9 μM). It is worth mentioning that compound **12** exhibited the most significant cytotoxicity against Hep-G2 and AGS (IC_50_ 5.1 and 2.7 *μ*M) and even better than that of oxaliplatin. As shown in **Fig. 2B**, after incubation with four human cancer cells for 48 h, extracts from two plants showed gradient increasing inhibitory activities with the increase of concentrations (0.1, 0.2, 0.4, 0.8, 1.6 mg/mL). As a result, the antitumor activity of extracts of *E. fischeriana* was significantly better than that of extracts of *E. ebracteolata* at various concentrations (P<0.05). At high concentration, the inhibition rate of extracts of *E. fischeriana* was equivalent to that of oxaliplatin.

Previous studies have shown that cancer, aging and other diseases are mostly related to the production of excessive free radicals [31]. Finding natural products with antioxidant activity from plants is a research hotspot, as plants contain a large number of small molecular compounds with antioxidant activity, such as polyphenols, vitamins, alkaloids, saponins, polysaccharides and so on [32]. The free radical scavenging activities of all isolated compounds, extracts of *E. fischeriana* and *E. ebracteolata*, along with vitamin C were evaluated by using ABTS and DPPH assay methods, respectively. The results of compounds **1−12** showed that compound **4** showed stronger (P<0.01) free radical scavenging activities than that of vitamin C because of its lower IC_50_ values (5.8 and 4.7 μg/mL in ABTS and DPPH assays, respectively). In addition, compound **1** showed strong free radical scavenging activity in DPPH analysis with a IC_50_ value of 11.1±0.8 μg/mL (P < 0.05). The results of ABTS and DPPH assay (**Fig. 3C and D**) revealed that free radical scavenging activities of extracts of *E. ebracteolata* (IC_50_=26.4±2.4, 32.7±1.0 μg/mL, respectively) was significantly (P< 0.01) better than that of extracts of *E. fischeriana* (IC_50_=43.4±0.7, 45.5±2.9 μg/mL, respectively).

In the folk, *E. fischeriana* and *E. ebracteolata* have been used to treat sores, furuncles and carbuncles mainly due to their stronger antibacterial activities [33]. Therefore, the evaluation of antimicrobial activity is a key part of the quality control of the two species. The minimal inhibitory concentration (MIC) of compounds **1−12** was tested by broth dilution method. As shown in **table 2**, the antibacterial activities of compounds **6−9** were better than those of acetophenones (**1****−5**) and tigliane diterpenoids (**10−12**) with MIC ranging from 0.78 to 6.25 μg/mL. In addition, both extracts of *E. fischeriana* and *E. ebracteolata* showed more significant inhibitory activities against the four tested bacteria than berberine at the concentration of 500 μg/mL. Compared with penicillin, extracts of *E. fischeriana* showed better inhibitory activities against *E. coli* and *P. Aeruginosa*, while and extracts of *E. ebracteolate* showed better inhibitory activities against *S. aureus and B. subtilis* (P<0.05).

**3. Tables**

Table. S1 Identification of mass features detected in the extracts of *E. fischeriana* and *E. ebracteolata*.

| Peak no | Identification | *m/z* Quasi-molecular [M+H]^+^ | *m/z* Calculated [M+H]^+^ | Δ ppm^a^ | Rt^b^ (min) | Main fragments *m/z* | Origin | Molecular formula | Level | Reference |
| --- | --- | --- | --- | --- | --- | --- | --- | --- | --- | --- |
| 1 | 6-hydroxy-2-methoxy-4-*O*-*α*-*L*-arabinofuranosyl-(1→6)-*β*-*D*-glucopyranoside | 477.1555 | 477.1608 | 11 | 6.44 | 345.1, 183.1 | EE^c^; EF^d^ | C_20_H_28_O_13_ | 1 | [5] |
| 2 | 6-hydroxy-2-methoxy-4-*O*-*β*-*D*-xylopyranosyl-(1→6)-*β*-*D*-glucopyranoside | 477.1556 | 477.1608 | 11 | 6.74 | 345.1, 183.1 | EE | C_20_H_28_O_13_ | 2 | [6] |
| 3 | 2,4-dihydroxy-6-methoxyacetophenoe-4-*O*-*β*-*D*-glucopyranoside | 345.1146 | 345.1186 | 12 | 7.17 | 183.1 | EE; EF | C_15_H_20_O_9_ | 2 | [7] |
| 4 | 2, 4-dihydroxy-6-methoxy-3-methylacetophenoe-4-*O*-*β*-*D*-glucopyranoside | 359.1313 | 359.1342 | 8 | 7.21 | 280.1, 197.1 | EE | C_16_H_22_O_9_ | 1 | [6] |
| 5 | 12-deoxyphorbaldehyde-13-acetate | 389.1922 | 389.1964 | 11 | 7.29 | 371.2, 311.2, 293.2 | EF | C_22_H_28_O_6_ | 2 | [8] |
| 6 | isomer of jolkinolide B | 331.1869 | 331.1909 | 12 | 7.47 | 313.2, 295.2, 271.2 | EF | C_20_H_26_O_4_ | 3 | −^e^ |
| 7 | ebractenoid L | 331.1870 | 331.1909 | 12 | 8.84 | 313.2, 295.2, 285.2, 271.2 | EE | C_20_H_26_O_4_ | 2 | [9] |
| 8 | 2, 4-dihydroxy-6-methoxy-3-methylacetophenoe-2-*O*-*β*-*D*-glucopyranoside | 358.1315 | 358.1342 | 8 | 9.05 | 280.1, 197.1 | EE | C_16_H_22_O_9_ | 2 | [3] |
| 9 | 3-oxo-12,18-ursadien-28-oic acid | 453.3385 | 453.3369 | 4 | 9.81 | 419.3, 357.2 | F | C_30_H_44_O_3_ | 2 | [10] |
| 10 | 6-hydroxy-2-methoxyacetophenone-4-*O*-(4'-galloyl)-*β*-*D*-glucopyranoside | 497.1250 | 497.1295 | 9 | 10.03 | 279.1, 183.1 | EE; EF | C_22_H_24_O_13_ | 2 | [11] |
| 11 | 2,4-dihydroxy-6-methoxyacetophenoe | 183.0638 | 183.0657 | 10 | 11.43 | 165.1 | EE; EF | C_9_H_10_O_4_ | 2 | [12] |
| 12 | fischerosides A | 553.2658 | 553.2649 | 2 | 12.07 | 313.2, 295.2, 277.2, 267.2 | EF | C_28_H_40_O_11_ | 1 | [13] |
| 13 | prostratin-20-*O*-(3'-galloyl)-*β*-*D*-glucopyranoside | 705.2696 | 705.2758 | 9 | 12.94 | 315.1, 313.2, 295.2, 249.6 | EF | C_35_H_44_O_15_ | 2 | [13] |
| 14 | 17-acetoxyjolkinolide A | 373.1980 | 373.2015 | 9 | 14.77 | 331.2, 313.2, 295.2, 277.2, 267.2 | EF | C_22_H_28_O_5_ | 1 | [14] |
| 15 | 2,4-dihydroxy-6-methoxy-3-methylacetophenoe | 197.0789 | 197.0814 | 13 | 14.91 | 179.1, 167.0, 155.0 | EE; EF | C_10_H_12_O_4_ | 1 | [15] |
| 16 | euphonoid D | 303.2292 | 303.2324 | 11 | 17.9 | 285.2, 267.2, 241.6 | EE | C_20_H_30_O_2_ | 2 | [16] |
| 17 | ent-16*α*,17-dihydroxyatisan-3-one | 321.2397 | 321.2430 | 10 | 18.2 | 303.2, 285.2 | EF | C_20_H_32_O_3_ | 2 | [17] |
| 18 | eupholides C | 379.2088 | 379.2121 | 9 | 19.49 | 361.2, 343.2, 295.2, | EF | C_21_H_30_O_6_ | 2 | [7] |
| 19 | tetrahydrojolkinolide B | 335.2167 | 335.2222 | 16 | 21.11 | 313.2, 295.2, 277.2 | EE | C_20_H_30_O_4_ | 2 | [18] |
| 20 | isomer of jolkinolide A | 315.1927 | 315.1960 | 10 | 21.62 | 297.2, 287.2 | EF | C_20_H_26_O_3_ | 3 | − |
| 21 | 17-hydroxyjolkinolide B | 347.1815 | 347.1858 | 12 | 22.75 | 329.2, 311.2, 301.2, 283.2, 273.2 | EF | C_20_H_26_O_5_ | 2 | [16] |
| 22 | 11*β*-hydroxy-8,14-epoxy-ent-abieta-13(15)-en-16,12-olide | 333.2034 | 333.2066 | 10 | 23.16 | 315.2, 297.2, 287.2 | EF | C_20_H_28_O_4_ | 2 | [19] |
| 23 | triptophenolide | 313.1768 | 313.1804 | 11 | 23.60 | 295.2, 285.2, 277.2 | EF | C_20_H_24_O_3_ | 2 | [20] |
| 24 | jolkinolide E | 301.2141 | 301.2168 | 9 | 23.99 | 283.2, 255.2 | EE | C_20_H_28_O_2_ | 2 | [8] |
| 25 | *Ent*-13(*R*)-hydroxy-3,14-dioxo-16-atisene | 317.2081 | 317.2117 |  | 25.19 | 299.2, 219.2 | EE | C_20_H_28_O_3_ | 2 | [21] |
| 26 | jolkinolide B | 331.1869 | 331.1909 | 12 | 26.20 | 313.2, 295.2, 285.2 | EE; EF | C_20_H_26_O_4_ | 1 | [22] |
| 27 | 3, 3'-diacetyl-4, 4'-dimethoxy-2, 2', 6, 6'-tetrahydroxy diphenylmethane | 377.1206 | 377.1236 | 8 | 26.58 | 195.1 | EE | C_19_H_20_O_8_ | 2 | [23] |
| 28 | methyl 7,11-dioxopimar-8-en-18-oate | 347.2184 | 347.2222 | 11 | 27.14 | 329.2, 315.2, 297.2, 287.2 | EF | C_21_H_30_O_4_ | 2 | [24] |
| 29 | 11*β*-hydroxy-*ent*-abieta-8(14), 13(15)-dien-16, 12β-olide | 317.2080 | 317.2117 | 12 | 27.79 | 299.2, 281.2, 271.2 | EE; EF | C_20_H_28_O_3_ | 2 | [9] |
| 30 | jolkinolide A | 315.1927 | 315.1960 | 10 | 28.6 | 297.2, 287.2 | EE, EF | C_20_H_26_O_3_ | 2 | [25] |
| 31 | *ent*-atis-16(17)-ene-3,14-dione | 301.2140 | 301.2168 | 9 | 31.27 | 283.2, 255.2 | EF | C_20_H_28_O_2_ | 2 | [26] |

^a^ Δ ppm: parts per million; ^b^ Rt: retention time; ^c^ EE: *Euphorbia ebracteolata* Hayata; ^d^ EF: *Euphorbia fischeriana* Steud; ^e^−: no data.

Table. S2 The key components were screened according to the condition of variable importance value (VIP) > 1.2 and fold change (FC) > 2.0.

| Peaks | Name | Fold Change | log_2_(FC) | VIP |
| --- | --- | --- | --- | --- |
| Peak 12^b^ | fischerosides A | 4483.1 | 12.13 | 2.4131 |
| Peak 19^a^ | tetrahydrojolkinolide B | 93.739 | 6.5506 | 1.7396 |
| Peak 15^a^ | 2,4-dihydroxy-6-methoxy-3-methylacetophenoe | 70.17 | 6.1328 | 1.653 |
| Peak 14^b^ | 17-acetoxyjolkinolide A | 61.489 | 5.9423 | 1.6691 |
| Peak 13^b^ | prostratin-20-*O*-(3'-galloyl)-*β*-*D*-glucopyranoside | 55.332 | 5.79 | 1.6509 |
| Peak 13^b^ | jolkinolide E | 55.332 | 5.79 | 1.6109 |
| Peak 24^a^ | methyl 7,11-dioxopimar-8-en-18-oate | 42.544 | 5.4109 | 1.439 |
| Peak 27^a^ | 3, 3'-diacetyl-4, 4'-dimethoxy-2, 2', 6, 6'-tetrahydroxy diphenylmethane | 19.126 | 4.2575 | 1.4255 |

^a^ Direction of comparison: *Euphorbia ebracteolata Hayata* / *Euphorbia fischeriana Steud*

^b^ Direction of comparison: *Euphorbia fischeriana Steud* / *Euphorbia ebracteolata Hayata*

Table. S3 Collecting information of 18 batches of different varieties of *E. fischeriana* and *E. ebracteolata*.

| Sample | Place of collection | Varieties | Medicinal part |
| --- | --- | --- | --- |
| EF-1 | Anhui | *E. fischeriana* | root |
| EF-2 | Anhui | *E. fischeriana* | root |
| EF-3 | Anhui | *E. fischeriana* | root |
| EF-4 | Liaoning | *E. fischeriana* | root |
| EF-5 | Liaoning | *E. fischeriana* | root |
| EF-6 | Liaoning | *E. fischeriana* | root |
| EF-7 | Henan | *E. fischeriana* | root |
| EF-8 | Henan | *E. fischeriana* | root |
| EF-9 | Henan | *E. fischeriana* | root |
| EE-1 | Anhui | *E. ebracteolata* | root |
| EE-2 | Anhui | *E. ebracteolata* | root |
| EE-3 | Anhui | *E. ebracteolata* | root |
| EE-4 | Liaoning | *E. ebracteolata* | root |
| EE-5 | Liaoning | *E. ebracteolata* | root |
| EE-6 | Liaoning | *E. ebracteolata* | root |
| EE-7 | Henan | *E. ebracteolata* | root |
| EE-8 | Henan | *E. ebracteolata* | root |
| EE-9 | Henan | *E. ebracteolata* | root |

**4. Figures**


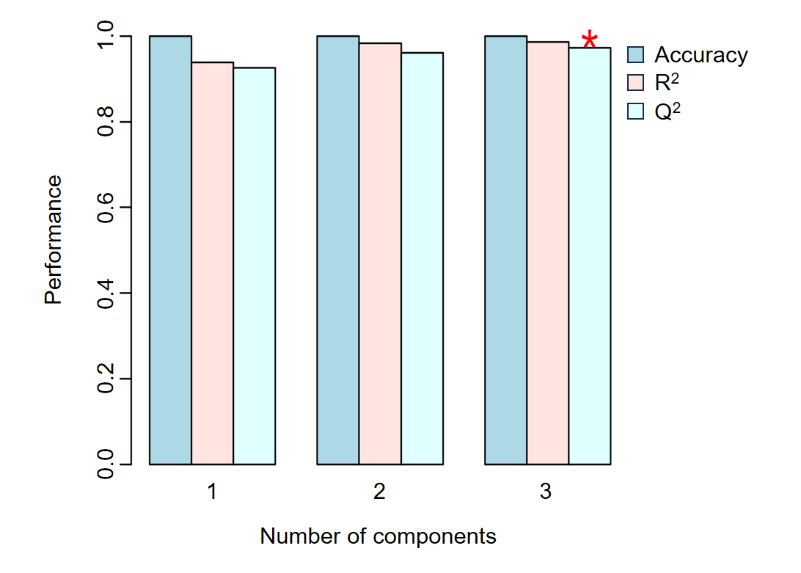


Fig. S1 The partial least-squares discriminant analysis (PLS-DA) model overview of different numbers of principal components

**
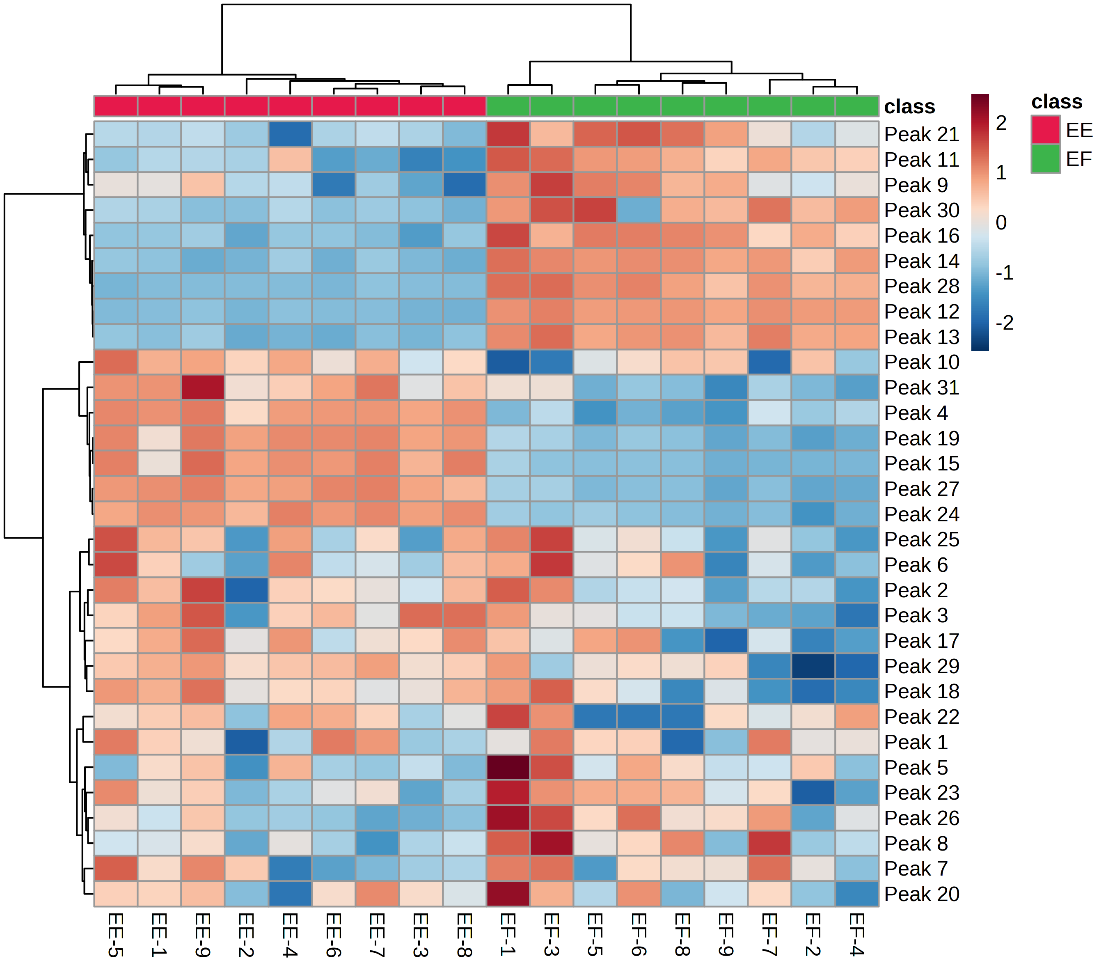
**

Fig. S2 The heat map of metabolites **1−31** from both the species. EE: *Euphorbia ebracteolata* Hayata*.* EF: *Euphorbia fischeriana* Steud.


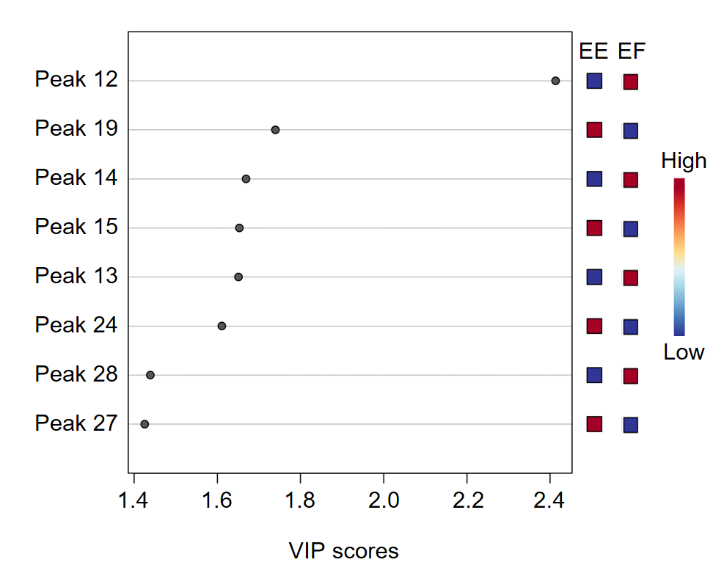


Fig. S3 Chemical markers with variable importance value (VIP)values larger than 1.2. EE: *Euphorbia ebracteolata* Hayata*.* EF: *Euphorbia fischeriana* Steud.


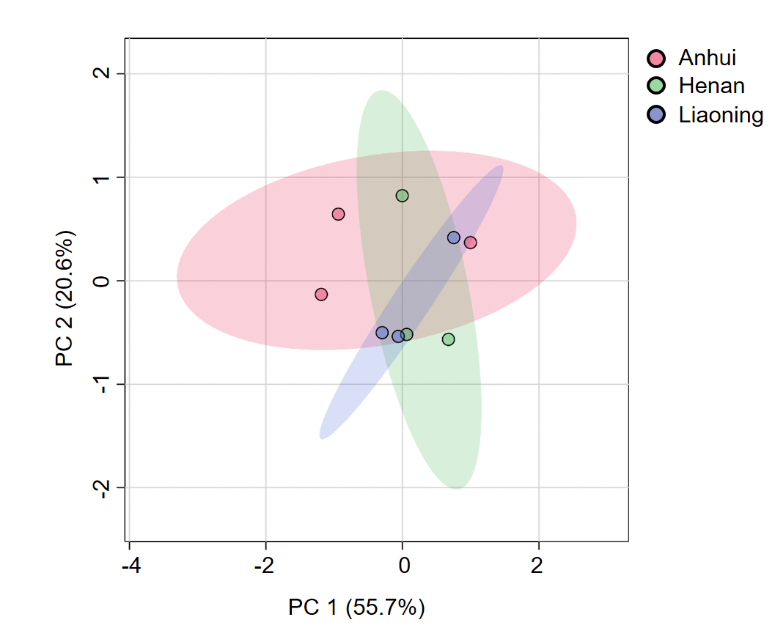


Fig. S4 The principal component analysis (PCA) Scores plot of *Euphorbia fischeriana* Steud


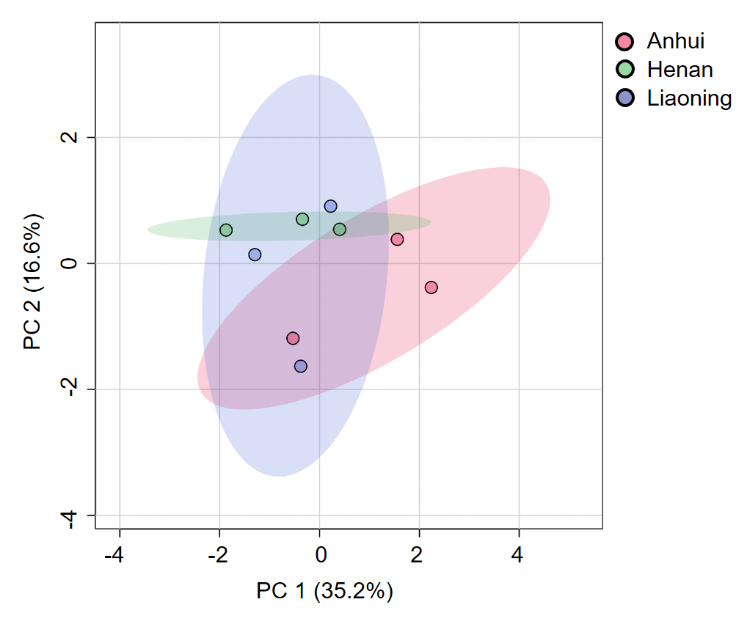


Fig. S5 The principal component analysis (PCA) Scores plot of *Euphorbia ebracteolata Hayata*

Fig. S6 The isolation procedures of *Euphorbia fischeriana* Steud

Fig. S7 The isolation procedures of *Euphorbia ebracteolata Hayata*


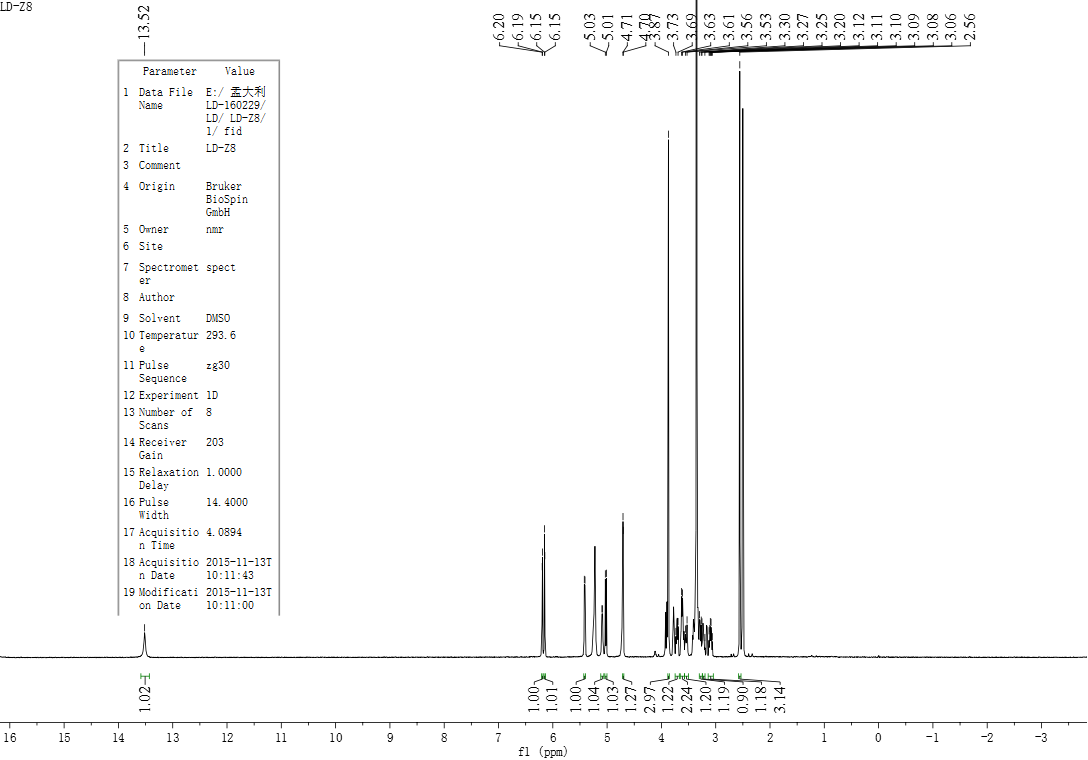


Fig. S8 The ^1^H-NMR spectrum of Comp. **1**


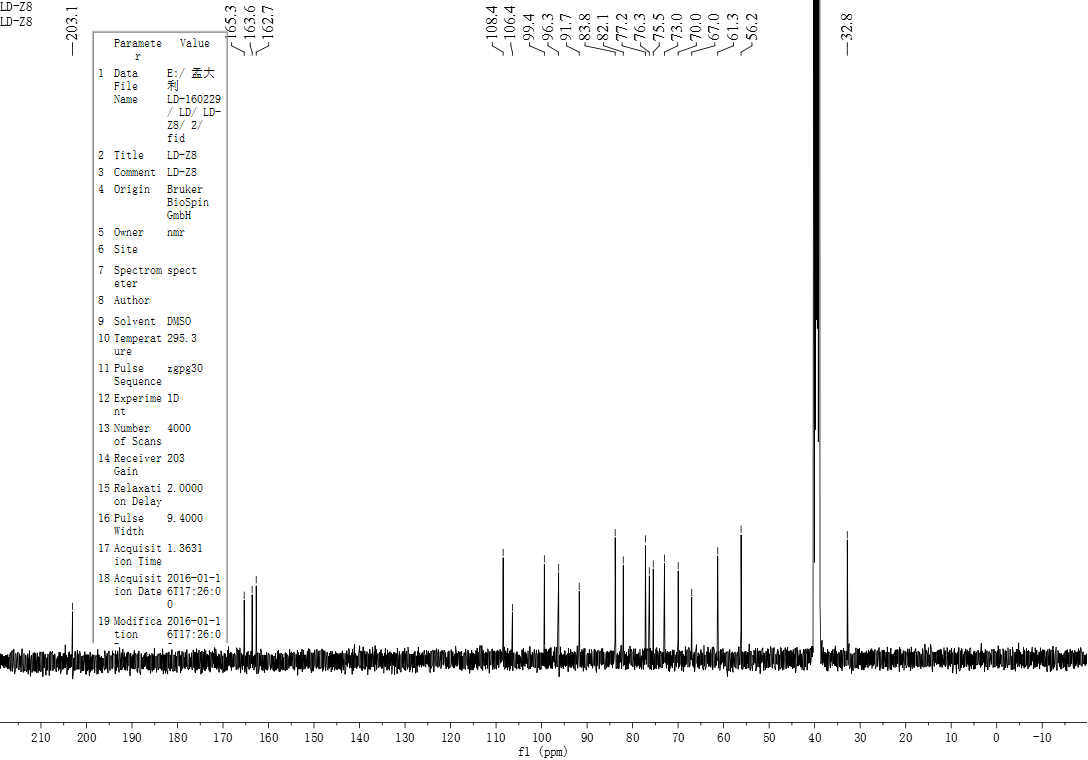


Fig. S9 The ^13^C-NMR spectrum of Comp. **1**


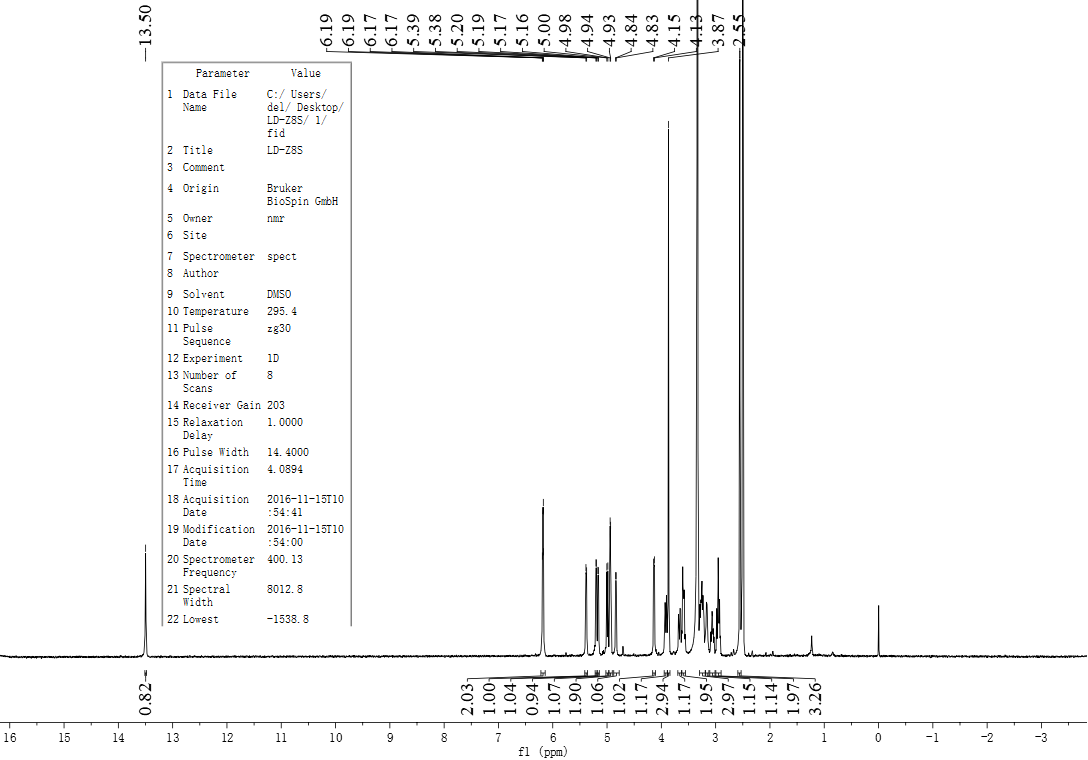


Fig. S10 The ^1^H-NMR spectrum of Comp. **2**


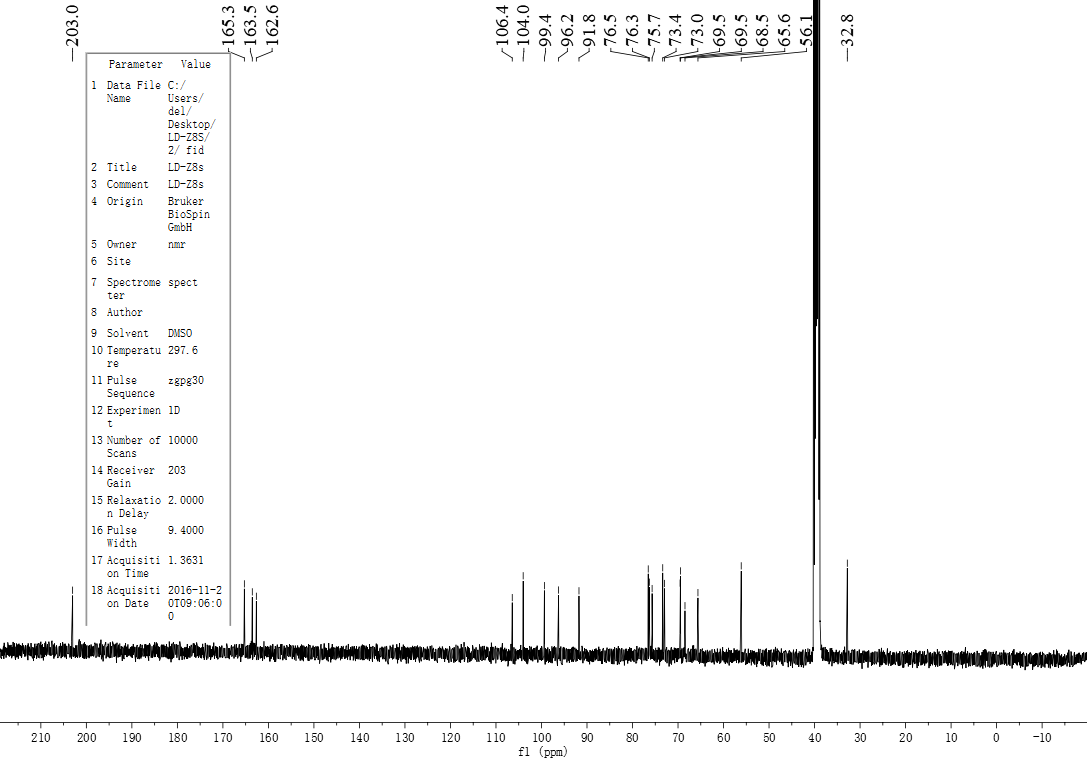


Fig. S11 The ^13^C-NMR spectrum of Comp. **2**


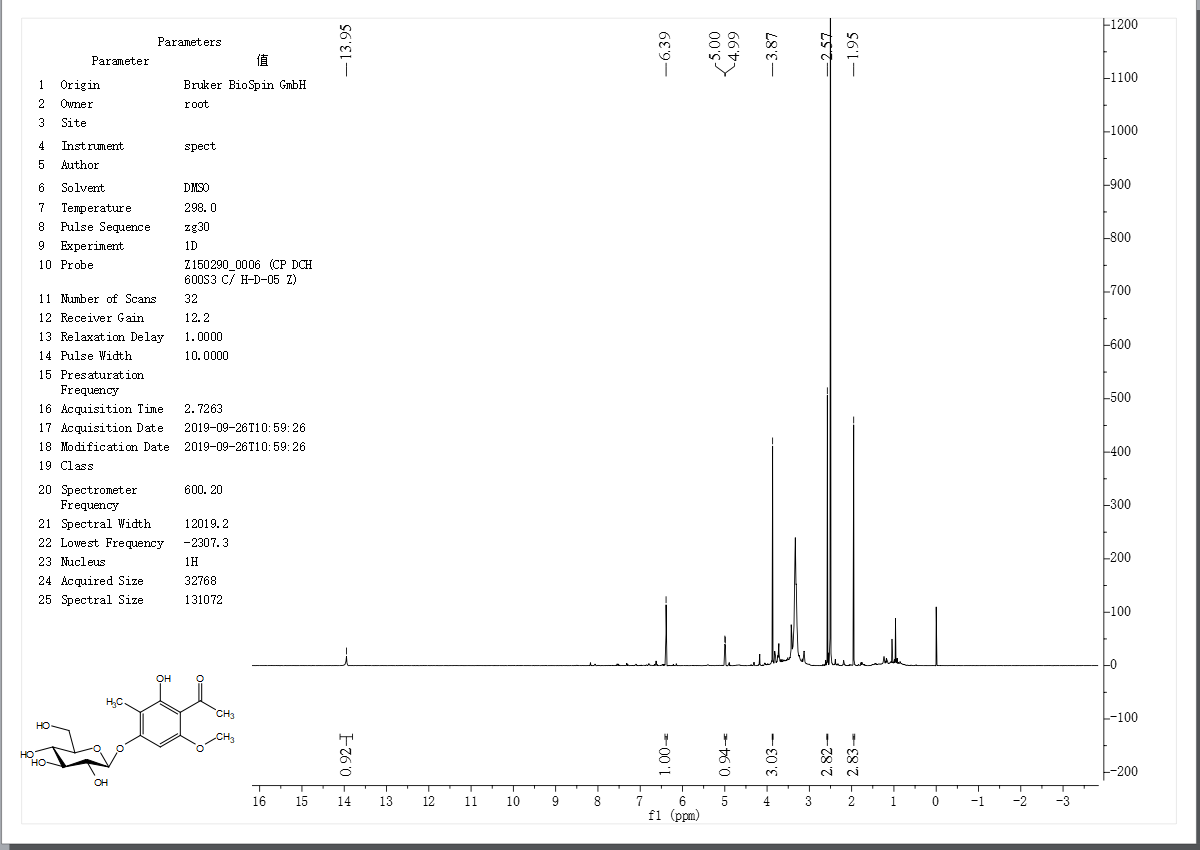


Fig. S12 The ^1^H-NMR spectrum of Comp. **3**


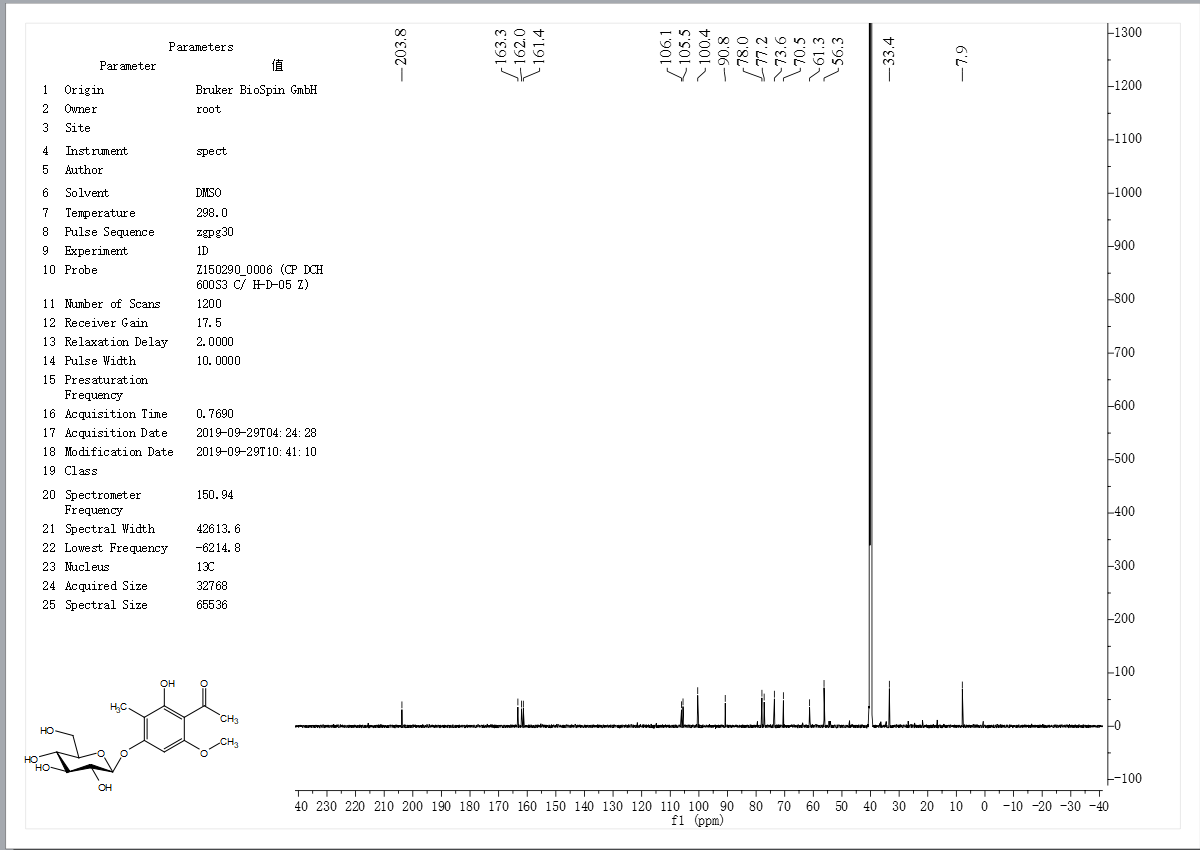


Fig. S13 The ^13^C-NMR spectrum of Comp. **3**


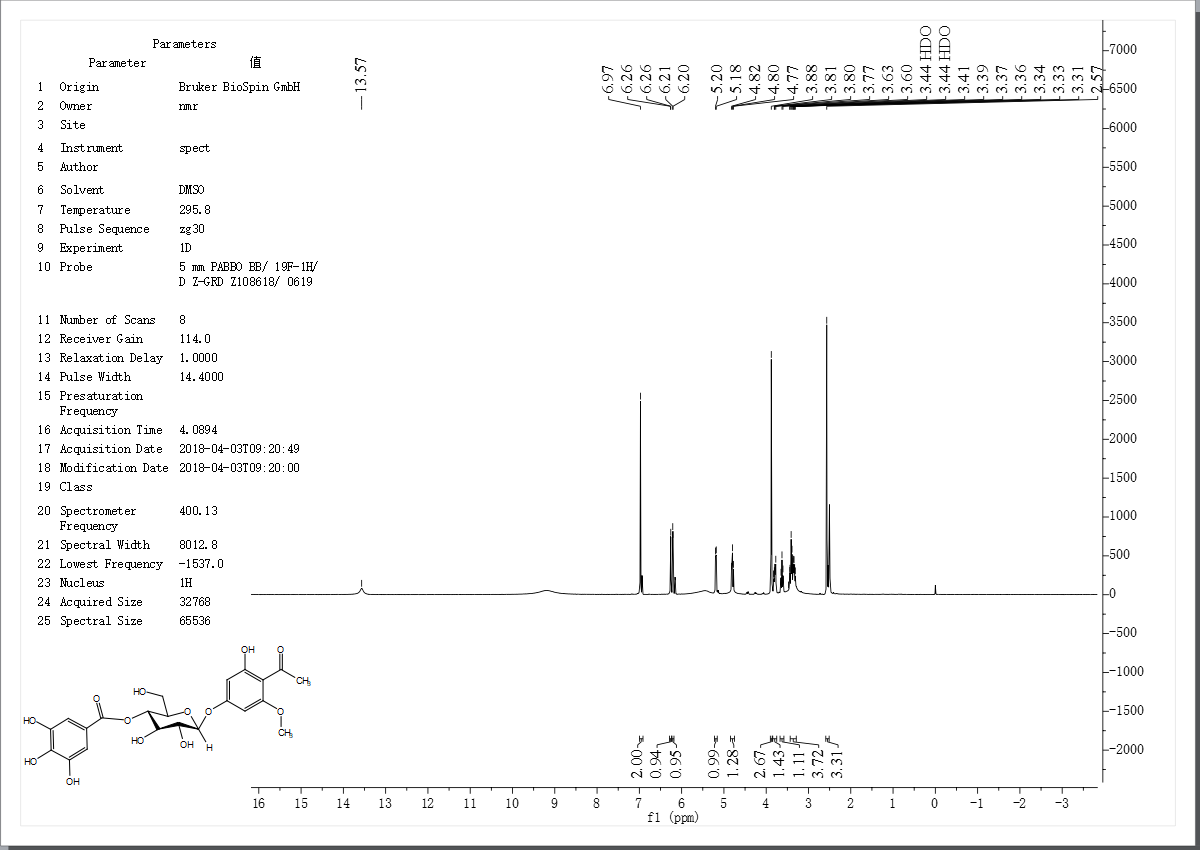


Fig. S14 The ^1^H-NMR spectrum of Comp. **4**


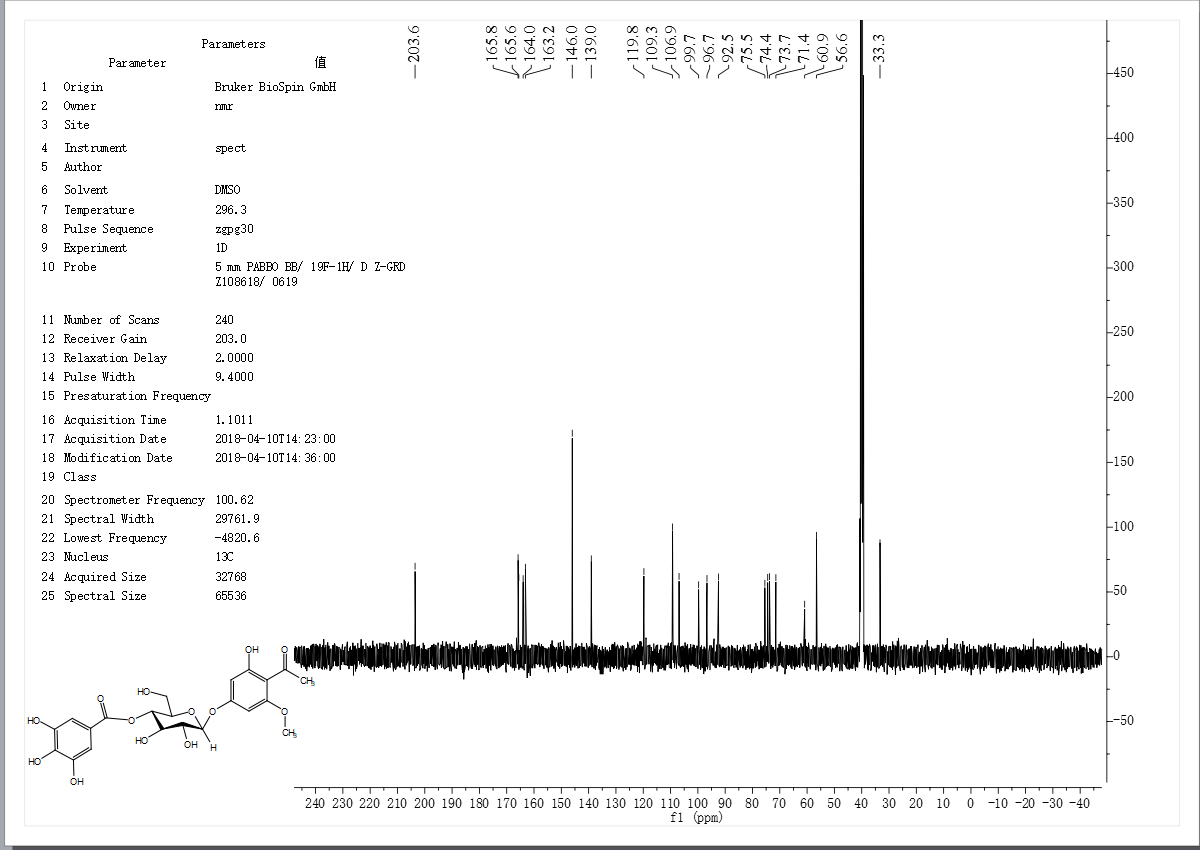


Fig. S15 The ^13^C-NMR spectrum of Comp. **4**


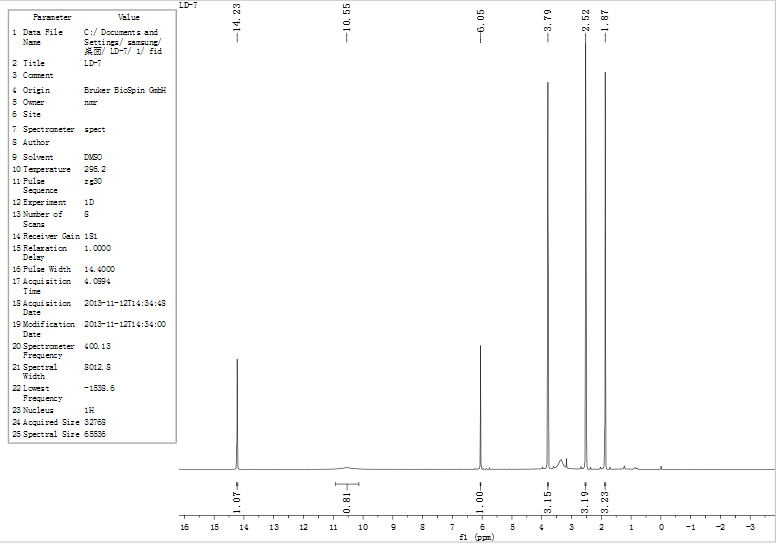


Fig. S16 The ^1^H NMR spectrum of Comp. **5**


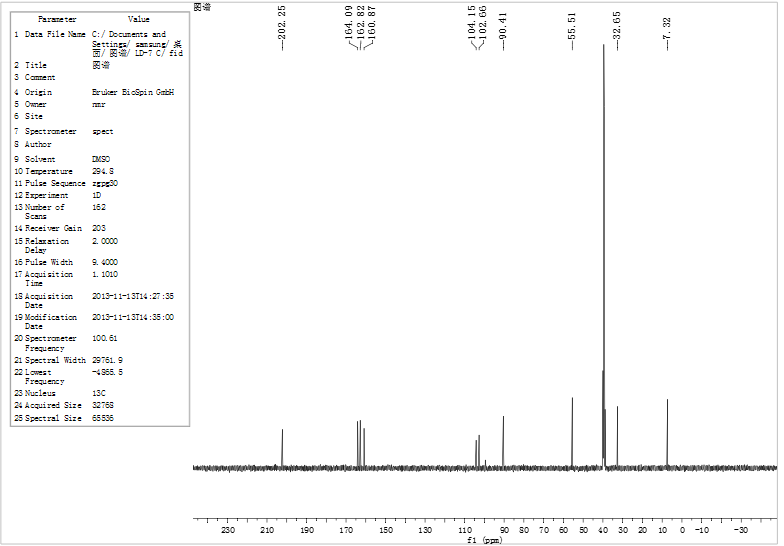


Fig. S17 The ^13^C NMR spectrum of Comp. **5**


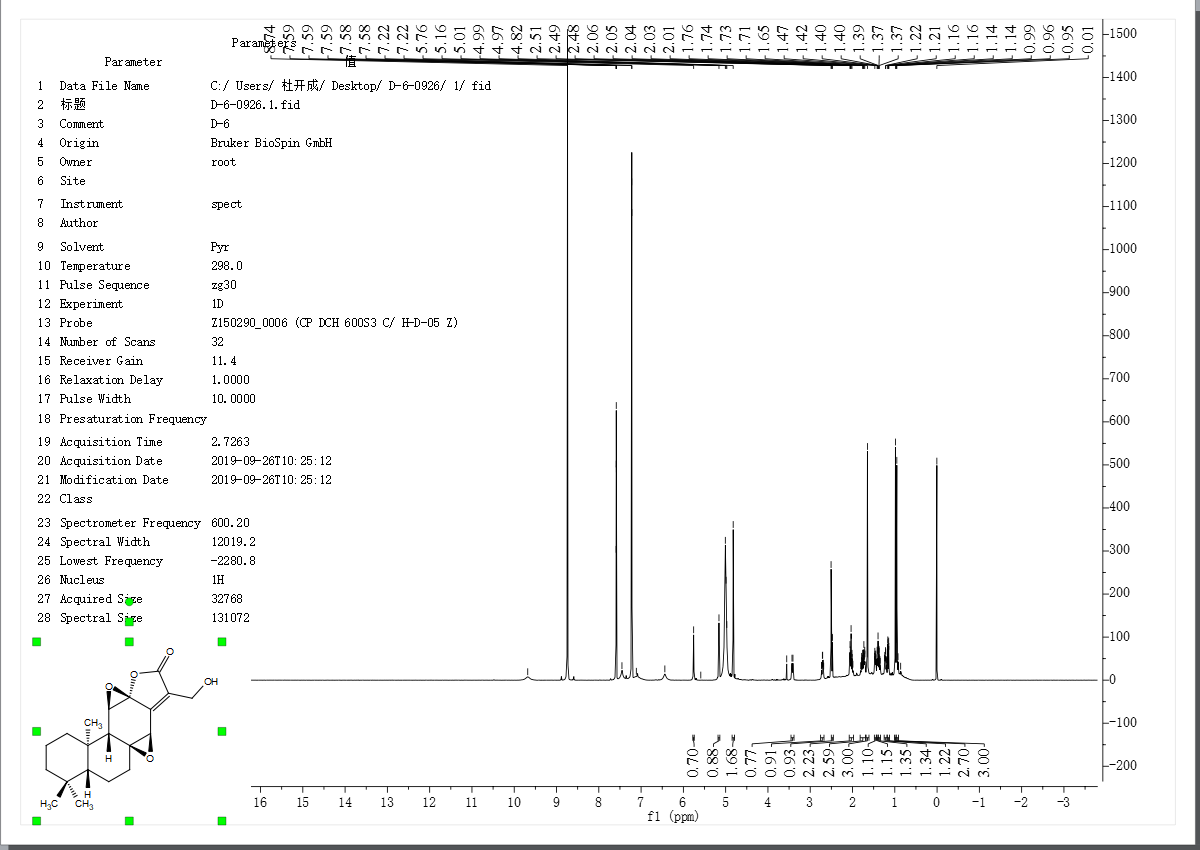


Fig. S18 The ^1^H-NMR spectrum of Comp. **6**


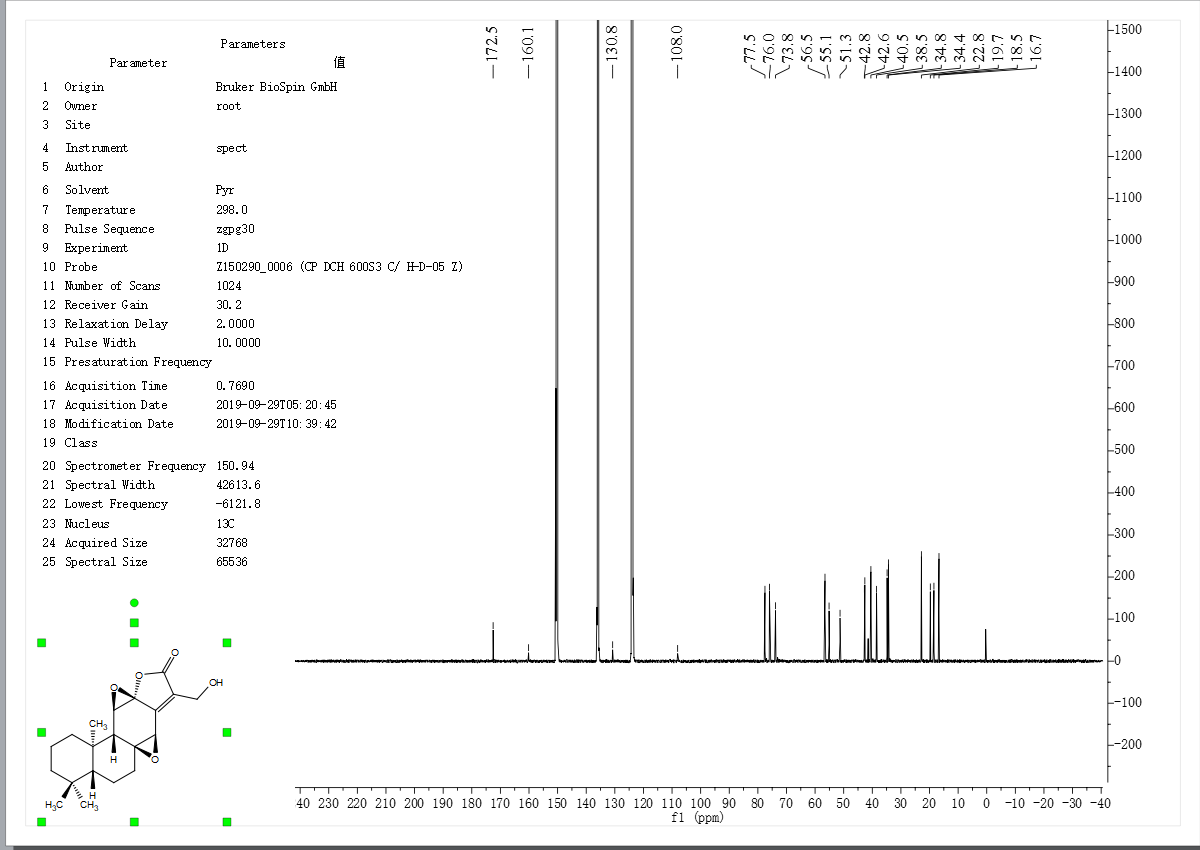


Fig. S19 The ^13^C-NMR spectrum of Comp. **6**


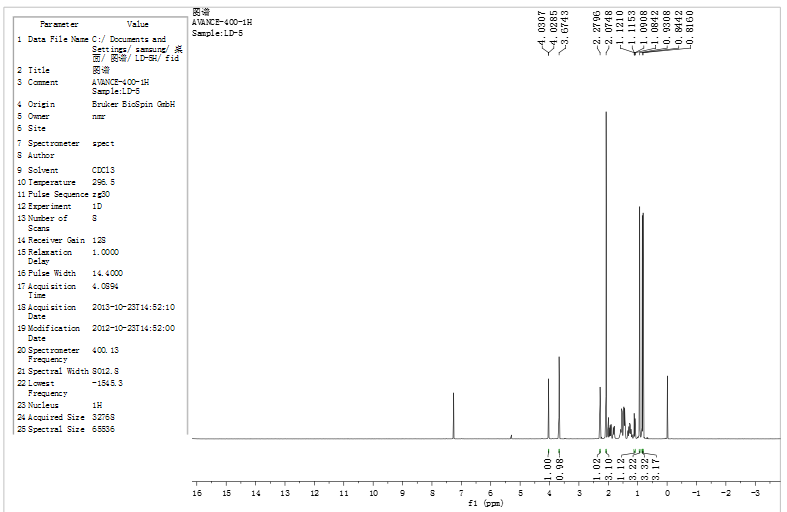


Fig. S20 The ^1^H NMR spectrum of Comp. **7**


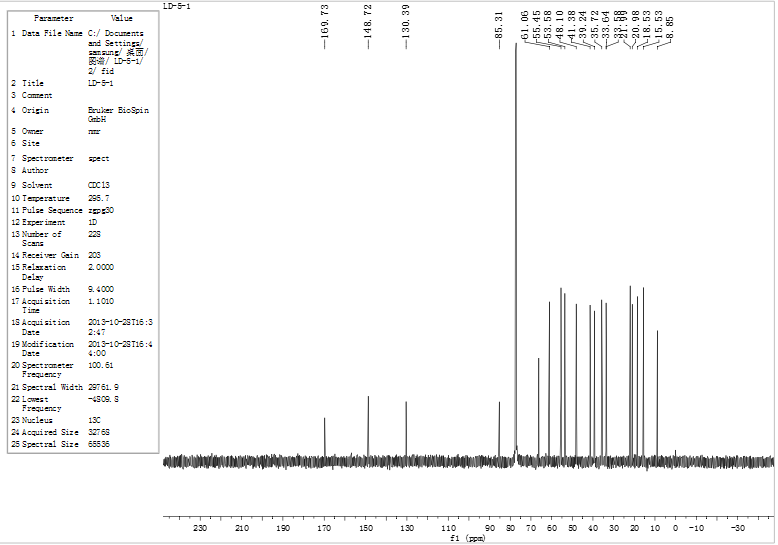


Fig. S21 The ^13^C NMR spectrum of Comp. **7**


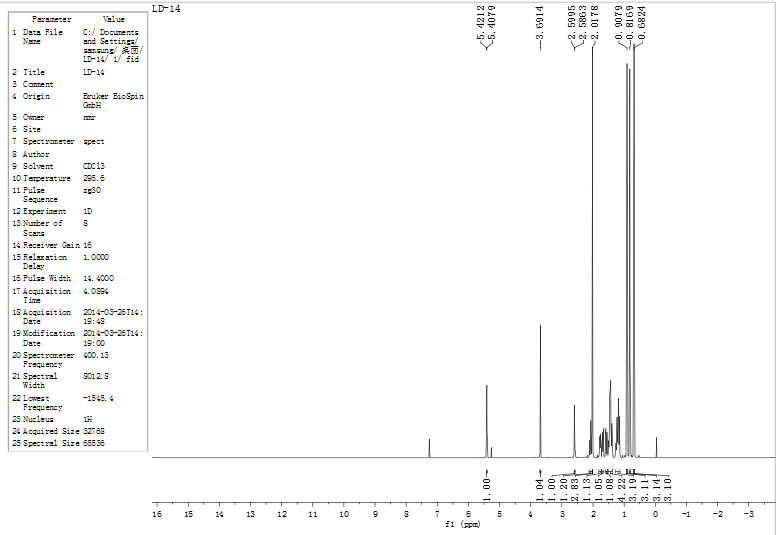


Fig. S22 The ^1^H NMR spectrum of Comp. **8**


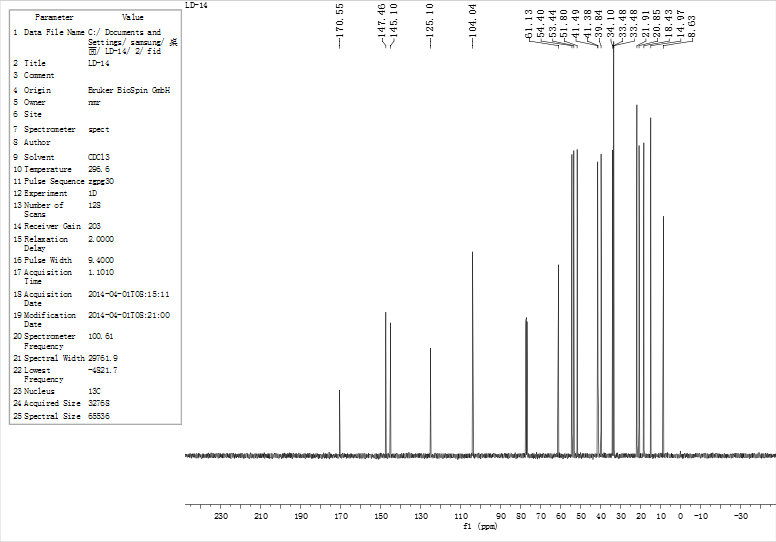


Fig. S23 The ^13^C NMR spectrum of Comp. **8**


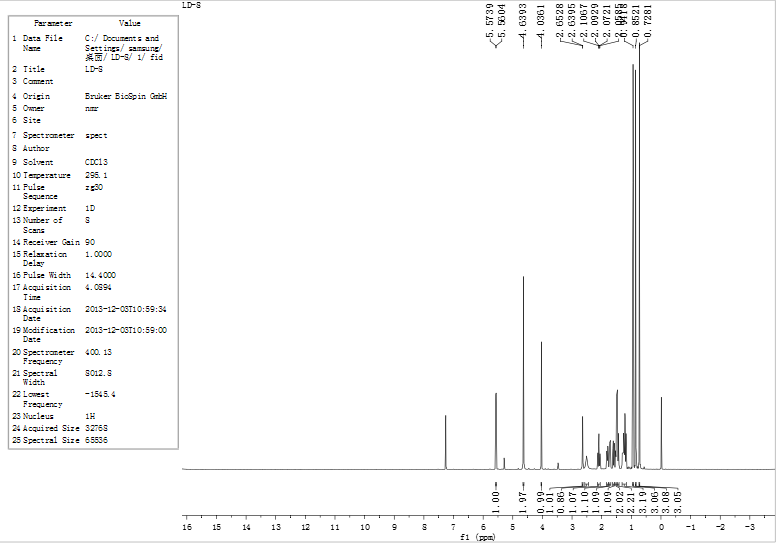


Fig. S24 The ^1^H NMR spectrum of Comp. **9**


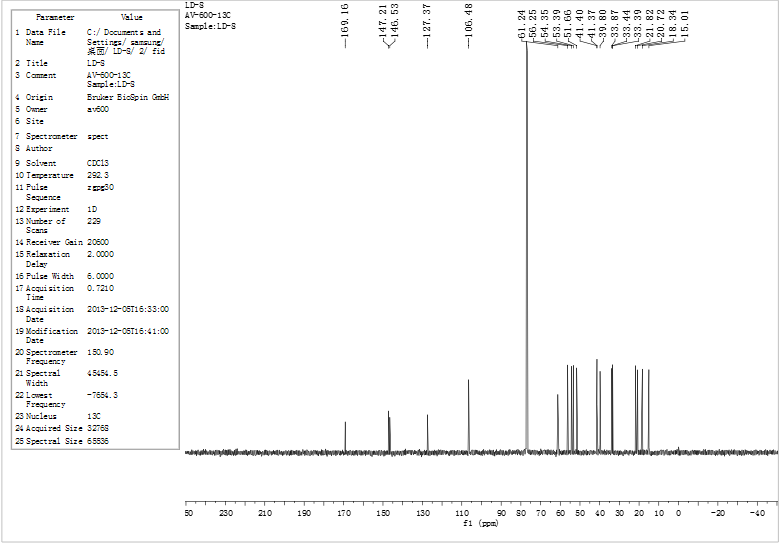


Fig. S25 The ^13^C NMR spectrum of Comp. **9**


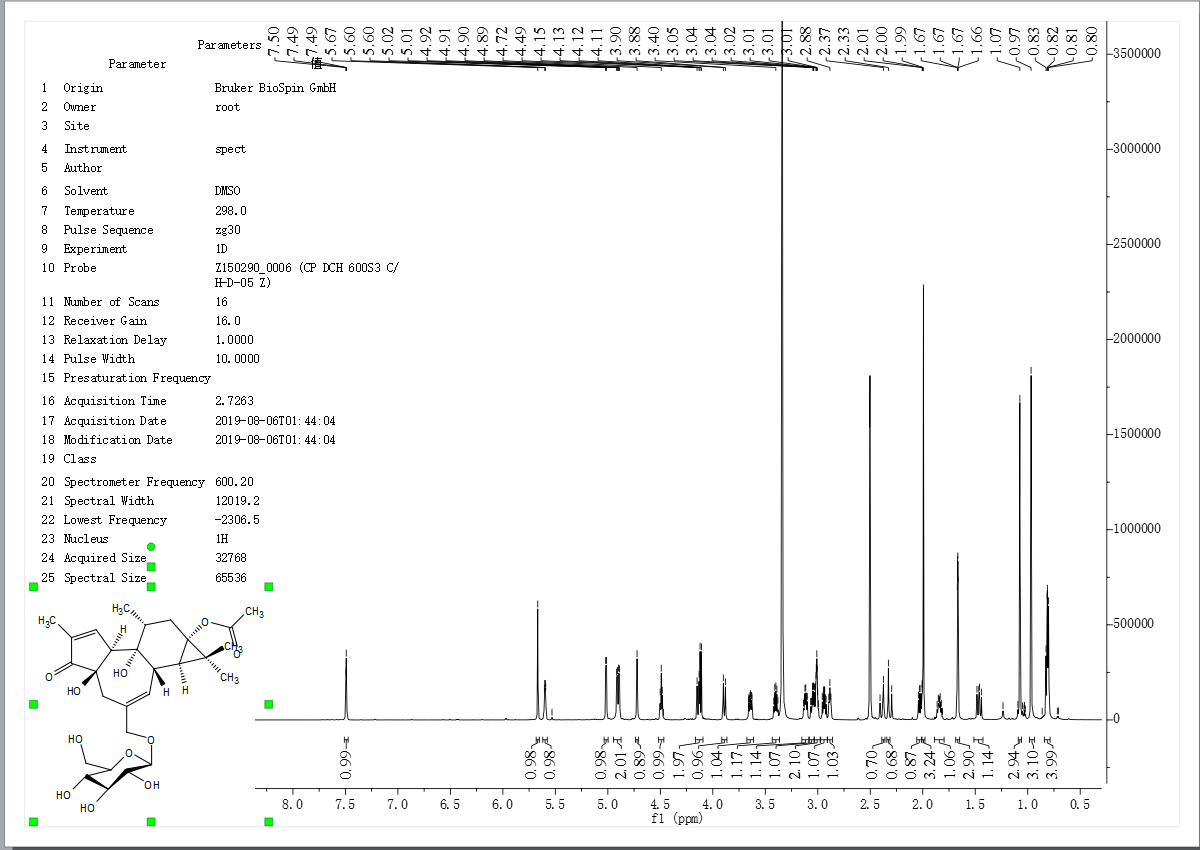


Fig. S26 The ^1^H-NMR spectrum of Comp. **10**


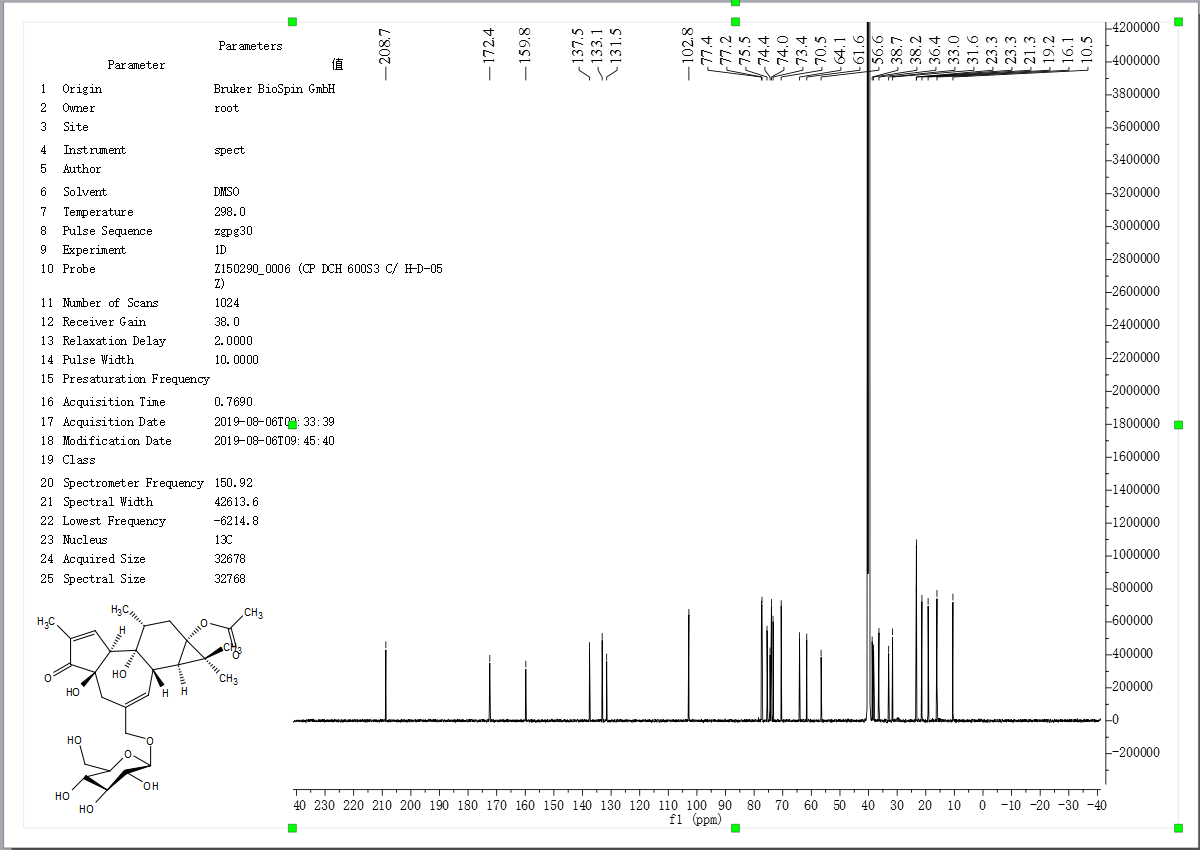


Fig. S27 The ^13^C-NMR spectrum of Comp. **10**


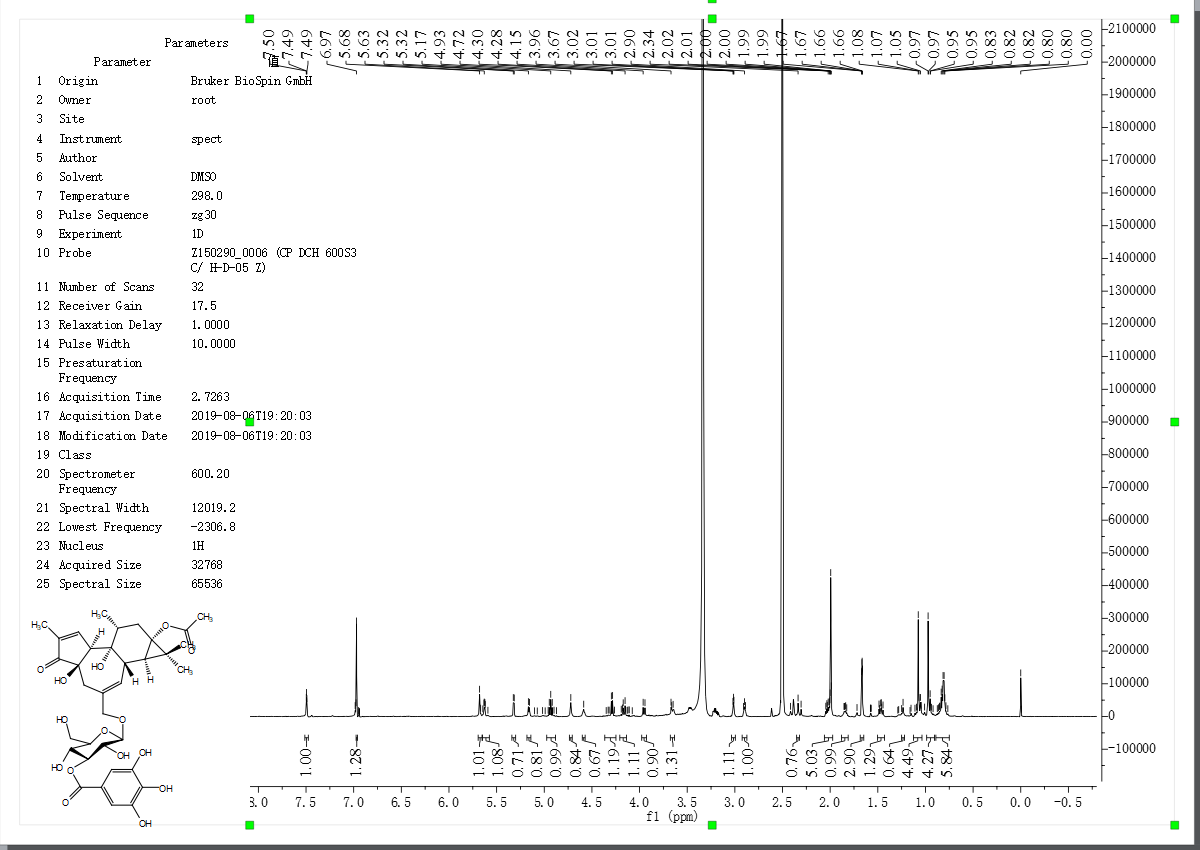


Fig. S28 The ^1^H-NMR spectrum of Comp. **11**


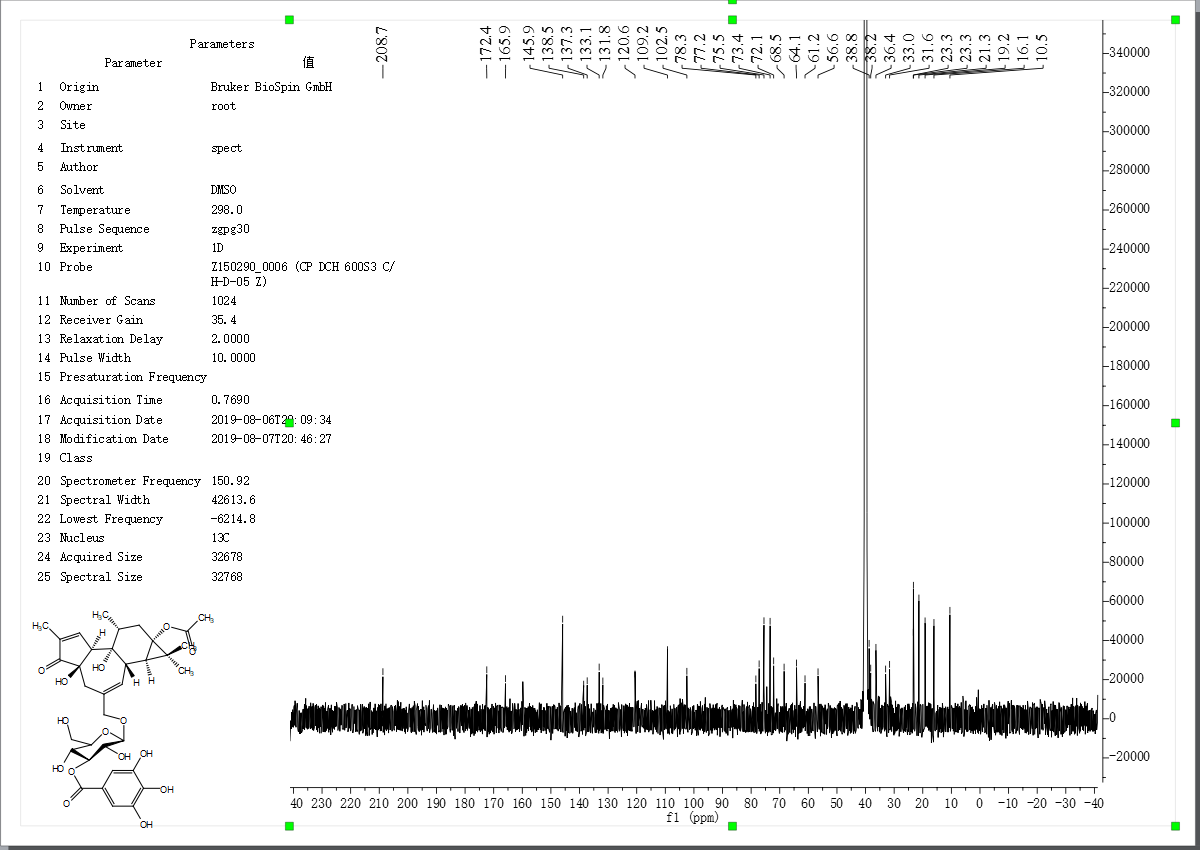


Fig. S29 The ^13^C-NMR spectrum of Comp. **11**


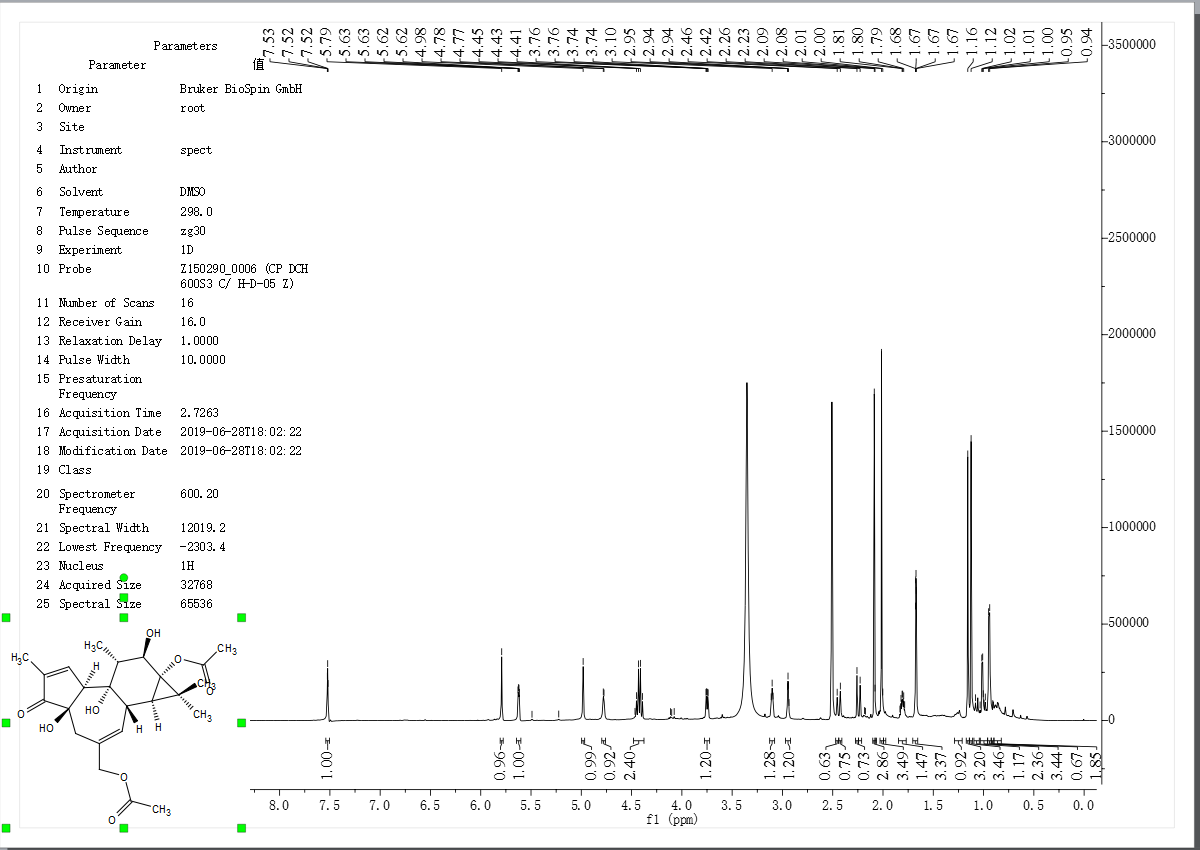


Fig. S30 The ^1^H-NMR spectrum of Comp. **12**


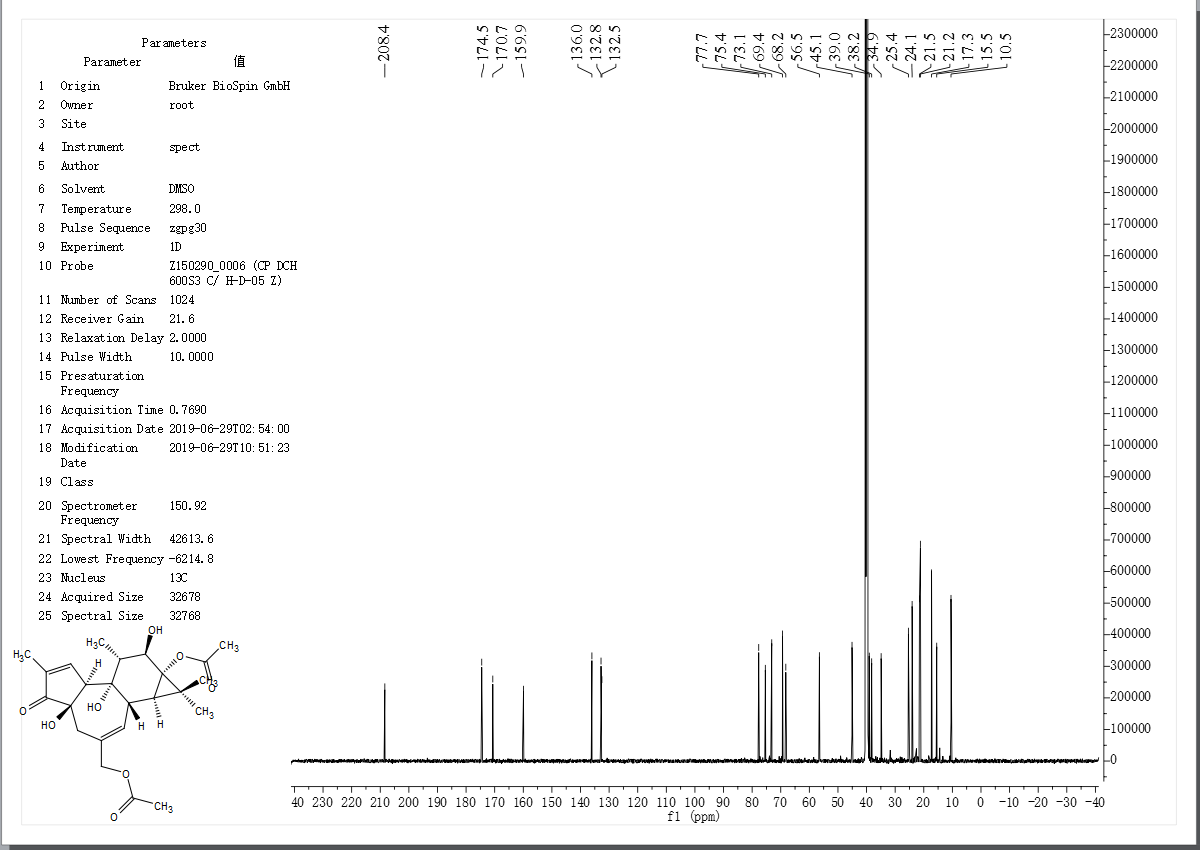


Fig. S31 The ^13^C-NMR spectrum of Comp. **12**


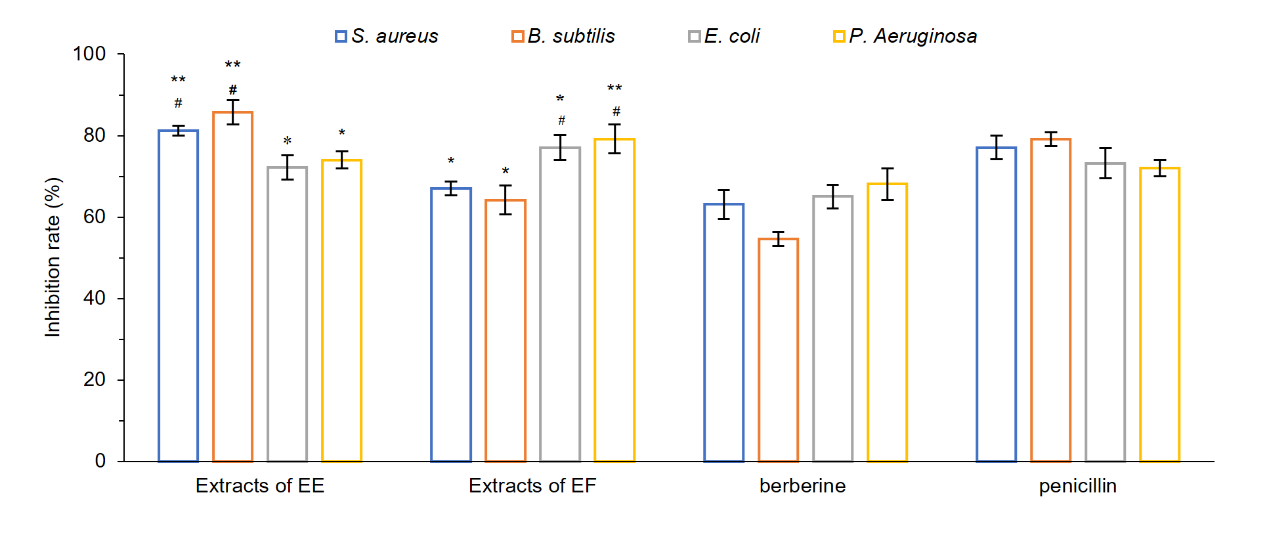


Fig. S32 Inhibitory rate of extracts from *Euphorbia fischeriana* Steud (EF) and *Euphorbia ebracteolata* Hayata (EE), berberine, and penicillin on *S. aureus, B. subtilis*, *E. coli* and *P. Aeruginosa* at 500 μg/mL. Data are presented as mean ± SD (n = 3) and analyzed by ANOVA. *P < 0.05 and **P < 0.01 compared with berberine group, ^#^P< 0.05 compared with penicillin group.

**5. References**

[1]. J. Guo, X. Feng, S. Zhou, et al., Potential anti-Alzheimer’s disease activities of the roots of Desmodium caudatum. Ind. Crop. Prod. 90 (2016) 94-99.

[2]. Y. Li, J. He, J. Zhang, et al., Existing knowledge on *Euphorbia fischeriana* Steud. (Euphorbiaceae): Traditional uses, clinical applications, phytochemistry, pharmacology and toxicology. J. Ethnopharmacol. 275 (2021), 114095.

[3]. T. Yang, J. He, Y. Yan, et al., *Euphorbia ebracteolata* Hayata (Euphorbiaceae): A systematic review of its traditional uses, botany, phytochemistry, pharmacology, toxicology, and quality control. Phytochemistry 186 (2021), 112736.

[4]. X. Liang, Z. G. Liu, Y. F. Cao, et al., Chemotaxonomic and chemical studies on two plants from genus of Euphorbia: *Euphorbia fischeriana* and *Euphorbia ebracteolata*. Biochem. Syst. Ecol. 57 (2014) 345-349.

[5]. S. S. El-Hawary, R. Mohammed, A. F. Tawfike, et al., Cytotoxic Activity and Metabolic Profiling of Fifteen Euphorbia Species. Metabolites 11 (2020) 15.

[6]. A. Wang, X. Gao, X. Huo, et al., Antioxidant acetophenone glycosides from the roots of *Euphorbia ebracteolata* Hayata. Nat. Prod. Res. 32 (2018) 2187-2192.

[7]. D. W. Li, X. P. Deng, X. He, et al., Eupholides AH, abietane diterpenoids from the roots of *Euphorbia fischeriana*, and their bioactivities. Phytochemistry 183 (2021), 112593.

[8]. C. Wang, X. Zhang, X. Yan, et al., Chemical profiling of *Euphorbia fischeriana* Steud. by UHPLC-Q/TOF-MS. J. Pharmaceut. Biomed. 151 (2018) 126-132.

[9]. Z. G. Liu, Z. L. Li, D. H. Li, et al., ent-Abietane-type diterpenoids from the roots of *Euphorbia ebracteolata* with their inhibitory activities on LPS-induced NO production in RAW 264.7 macrophages. Bioorg. Med. Chem. Lett. 26 (2016) 1-5.

[10]. L. Li, C. Muyan and B. Kenneth, Storey,. Metabolic response of longitudinal muscles to acute hypoxia in sea cucumber Apostichopus japonicus (Selenka): A metabolome integrated analysis. Comp. Biochem. Physiol. Part D Genomics Proteomics 29 (2019) 235-244.

[11]. S. S. Huang, P. Li, B. J. Zhang, et al., Acetophenone glycosides from the roots of *Euphorbia fischeriana* and their inhibitory effects against Mycobacterium smegmatis. Phytochemistry Lett. 19 (2017) 151-155.

[12]. H. Jun, K. Jie, J. Xu, et al., Fischeriana A, a meroterpenoid with an unusual 6/6/5/5/5/6/6 heptacyclic carbon skeleton from the roots of *Euphorbia fischeriana*. Org. Biomol. Chem. 17 (2019) 2721-2724.

[13]. C. Wang, Q. Yan, Y. Ma, et al., ent-Abietane and Tigliane Diterpenoids from the Roots of *Euphorbia fischeriana* and Their Inhibitory Effects against Mycobacterium smegmatis. J. Nat. Prod. 80 (2017) 1248-1254.

[14]. Y. Wang, R. Huang, H. Wang, et al., Diterpenoids from the Roots of *Euphorbia fischeriana*. J. Nat. Prod. 69 (2006) 967-970.

[15]. P. J. Hylands and K. Ingolfsdottir. The isolation of methyl β-orsellinate from Stereocaulon alpinum and comments on the isolation of 4,6-dihydroxy-2- methoxy-3-methylacetophenone from stereocaulon species. Phytochemistry 24 (1985) 127-129.

[16]. X. L. Yan, J. S. Zhang, J. L. Huang, et al., Euphonoids A-G, cytotoxic diterpenoids from *Euphorbia fischeriana*. Phytochemistry 166 (2019), 112064.

[17]. M. Wang, Q. Wang, Q. Wei, et al., Two new ent-atisanes from the root of *Euphorbia fischeriana* Steud. Nat. Prod. Res. 30 (2016) 144-149.

[18]. Y. Ma, X. Tang, W. Yuan, et al., Abietane Diterpernoids from the Roots of *Euphorbia ebracteolata*. Nat. Prod.Bioprosp. 8 (2018) 131-135.

[19]. W. Li, Y. Lin, Y. Wang, et al., Development of a Matrix Solid-Phase Dispersion Extraction Combined with UPLC/Q-TOF-MS for Determination of Phenolics and Terpenoids from the *Euphorbia fischeriana*. Molecules 22 (2017) 1524.

[20]. H. Xu, L. Liu, X. Fan, et al., Identification of a diverse synthetic abietane diterpenoid library for anticancer activity. Bioorg. Med. Chem. Lett. 27 (2017) 505-510.

[21]. B. Wang, Z. Dai, X. W. Yang, et al., Novel nor-monoterpenoid indole alkaloids inhibiting glioma stem cells from fruits of Alstonia scholaris. Phytomedicine 48 (2018) 170-178.

[22]. Y. Ren and A. D. Kinghorn. Development of Potential Antitumor Agents from the Scaffolds of Plant-Derived Terpenoid Lactones. J. Med. Chem. 63 (2020) 15410-15448.

[23]. M. Guang, Fu, Y. Bo, et al., Two novel phloroglucinol derivatives from *Euphorbia ebracteolata* hayata. J. Asian Nat. Prod. Res. 8 (2006) 149-153.

[24]. W. Herz and A. Hall. Resin acids. XXV. Chromic acid oxidation of .DELTA.8,9-primaranes and isopimaranes. Long range deshielding in 8,9 epoxides. J. Org. Chem. 39 (1974) <https://doi.org/10.1021/jo00915a00002>.

[25]. J. Zhang, J. He, X. Wang, et al., Ent-abietane diterpenoids and their probable biogenetic precursors from the roots of *Euphorbia fischeriana*. RSC Adv. 7 (2017) 55859-55865.

[26]. X. Kuang, W. Li, Y. Kanno, et al., ent-Atisane diterpenoids from *Euphorbia fischeriana* inhibit mammosphere formation in MCF-7 cells. J. Nat. Med. 70 (2016) 120-126.

[27]. R. M. Salek, C. Steinbeck, M. R. Viant, et al., The role of reporting standards for metabolite annotation and identification in metabolomic studies. GigaScience 2 (2013) 1-3.

[28]. L. W. Sumner, A. Amberg, D. Barrett, et al., Proposed minimum reporting standards for chemical analysis. Metabolomics 3 (2007) 211-221.

[29]. C. Han, Y. Peng, Y. Wang, et al., Cytotoxic ent-Abietane-type diterpenoids from the roots of *Euphorbia ebracteolata*. Bioorg. Chem. 81 (2018) 93-97.

[30]. T. Ma, Y. Sun, L. Liu, et al., Optimization of extraction for diterpenoids from *Euphorbia fischeriana* Steud using response surface methodology and structure identification by UPLC-Q-TOF-MS. Nat. Prod. Res. 35 (2021) 2458-2462.

[31]. Y. Shebis, D. Iluz, Y. Kinel-Tahan, et al., Natural Antioxidants: Function and Sources. Food Nutr. Sci. 04 (2013) 643-649.

[32]. M. S. F. Lie Ken Jie and M. Khysar Pasha. Fatty acids, fatty acid analogues and their derivatives. Nat. Prod. Rep. 15 (1998) 607-629.

[33]. B. Wang, P. Wei, S. Wan, et al., Ginkgo biloba exocarp extracts inhibit S. aureus and MRSA by disrupting biofilms and affecting gene expression. J. Ethnopharmacol. 271 (2021), 113895.
